# Minimum and optimal combined variations in sleep, physical activity, and nutrition in relation to all-cause mortality risk

**DOI:** 10.1186/s12916-024-03833-x

**Published:** 2025-02-26

**Authors:** Emmanuel Stamatakis, Nicholas A. Koemel, Raaj K. Biswas, Matthew N. Ahmadi, Margaret Allman-Farinelli, Stewart G. Trost, Elif Inan-Eroglu, Borja del Pozo Cruz, Yu Sun Bin, Svetlana Postnova, Mitch J. Duncan, Dorothea Dumuid, Helen Brown, Carol Maher, Luigi Fontana, Stephen Simpson, Peter A. Cistulli

**Affiliations:** 1 Mackenzie Wearables Research Hub, Charles Perkins Centre, The University of Sydney, Sydney, NSW Australia; 2 School of Health Sciences, Faculty of Medicine and Health, The University of Sydney, Sydney, NSW Australia; 3 Charles Perkins Centre, The University of Sydney, Sydney, NSW Australia; 4 Nutrition and Dietetics, School of Nursing, Faculty of Medicine and Health, The University of Sydney, Sydney, NSW Australia; 5 School of Human Movement and Nutrition Sciences, The University of Queensland, and Children’s Health Queensland, Brisbane, QLD Australia; 6 Department of Molecular Epidemiology, German Institute of Human Nutrition Potsdam-Rehbruecke, Nuthetal, Germany; 7 Faculty of Sports Sciences, Universidad Europea de Madrid, Madrid, Spain; 8 Faculty of Biomedical and Health Sciences, Universidad Europea de Madrid, Madrid, Spain; 9 Department of Sports Science and Clinical Biomechanics, University of Southern Denmark, Odense, Denmark; 10 Northern Clinical School, Faculty of Medicine and Health, University of Sydney, Sydney, Australia; 11 School of Physics, Faculty of Science, The University of Sydney, Sydney, NSW Australia; 12 School of Medicine and Public Health, College of Health, Medicine, and Wellbeing, The University of Newcastle, University Drive, Callaghan, NSW Australia; 13 Centre for Active Living and Learning, University of Newcastle, Callaghan, NSW Australia; 14 Alliance for Research in Exercise, Nutrition and Activity, Allied Health and Human Performance, University of South Australia, Adelaide, South Australia Australia; 15 School of Exercise and Nutrition Sciences, Faculty of Health, Deakin University, Geelong, VIC Australia; 16 Department of Endocrinology, Royal Prince Alfred Hospital, Sydney, NSW Australia; 17 School of Life and Environmental Sciences, Faculty of Science, The University of Sydney, Sydney, NSW Australia; 18 Department of Respiratory and Sleep Medicine, Royal North Shore Hospital, Sydney, NSW Australia

**Keywords:** Sleep, Nutrition, Physical activity, Exercise, Diet, Mortality, Cohort studies

## Abstract

**Background:**

Sleep, physical activity, and nutrition (SPAN) are critical behaviours for health, although they have traditionally been studied separately. We examined the combined associations of SPAN and the minimum between-individual variations associated with meaningfully lower all-cause mortality risk.

**Methods:**

This prospective cohort analysis included 59,078 participants from the UK Biobank (median age: 64.0 years; 45.4% male) who wore trackers for 7 days and self-reported dietary data. Wearable-measured sleep (hours/day) and moderate to vigorous physical activity (MVPA; mins/day) were calculated using a machine learning based schema. A 10-item diet quality score (DQS) assessed the intake of vegetables, fruits, fish, dairy, whole grains, vegetable oils, refined grains, processed and unprocessed meats, and sugary beverages (0–100 for all components with higher values indicating higher quality). Cox proportional hazards models were used to estimate hazard ratios (HR) for all-cause mortality risk across 27 separate joint tertile combinations of SPAN behaviours with the lowest tertile for all three as the referent group. For more granular clinical interpretations, we examined combined incremental dose–response changes of the SPAN behaviours using the 5th percentile of each behaviour as the referent point.

**Results:**

Over the 8.1-year median follow-up time, 2,458 mortality events occurred. Compared to the referent group of combined SPAN exposure (lowest tertiles for all three), the optimal SPAN combination involving moderate sleep duration (7.2–8.0 h/day), high MVPA (42–103 min/day), and a DQS between 57.5 and 72.5 was associated with an HR of 0.36 (95% CI: 0.26–0.50). Relative to the 5th percentile of sleep (5.5 h/day), physical activity (7.3 min/day), and nutrition (36.9 DQS), a theoretical minimum combined increase of 15 min/day of sleep, 1.6 min/day MVPA, and 5 DQS points (corresponding to e.g., extra 1/2 serving of vegetables per day or 1 less serving of processed meat per week) was associated with 10% lower all-cause mortality risk (0.90; 0.88–0.93). Combined increases of 75 min/day of sleep, 12.5 min/day MVPA, and 25 DQS points were associated with 50% lower all-cause mortality risk (0.50; 0.44–0.58).

**Conclusions:**

This study highlights the potential health value of subtle combined SPAN modification in relation to mortality risk and expands opportunities for more holistic recommendations.

**Supplementary Information:**

The online version contains supplementary material available at 10.1186/s12916-024-03833-x.

## Background

Adequate sleep, physical activity, and nutrition (SPAN) are vital for health and well-being, and each has established links with lower risks of chronic disease and premature mortality [1–3]. Insufficient sleep is associated with impaired metabolic and brain health through mechanisms such as insulin resistance, inflammation, and disruption of appetite hormones [4, 5]. Physical inactivity is a major contributor to the etiology of multiple chronic diseases [2]; adhering to current physical activity guidelines is linked with approximately 30–61% lower all-cause mortality risk [6, 7]. Excessive calorie intake and unhealthy dietary patterns play a key role in the pathogenesis of some of the most common non-communicable diseases [8, 9], premature mortality [10], and the biology of ageing itself [11, 12]. Sleep, physical activity, and nutrition are behaviourally interlinked, often clustering together to form broader lifestyle patterns [13, 14]. For instance, sleep deprivation can lead to lower physical activity due to fatigue, while exercise uptake may lead to improvements in sleep [15–17]. Poor sleep upregulates signaling for hunger hormones and downregulates satiety hormones, directly influencing food intake and food choices [18]. Additionally, low-quality diets negatively impact sleep by disrupting the neurotransmitters that regulate normal sleep–wake cycles [19]. Despite this high degree of behavioural interdependency, the three SPAN behaviours have been studied and promoted in narrow unidisciplinary silos, and research that examines the combined impact of these key chronic disease risk factors is scarce. A small number of studies have explored joint associations of pairs of these behaviours with all-cause, cardiovascular disease, and cancer mortality, for example diet and physical activity [20, 21], or sleep and physical activity [22, 23]. A study of just over 1000 National Health and Nutrition Examination Survey participants revealed the effects of different combinations of the SPAN exposures with all-cause mortality via model-based clustering analysis [24]. This study revealed unique behavioural SPAN profiles that were associated with mortality.

This emerging body of evidence suggests that addressing multiple SPAN behaviours concurrently may have promising potential for reducing mortality risk. Sustainable SPAN behaviour change is challenging, particularly through the traditional intervention approaches that usually set targets for substantial changes in one of the three SPAN behaviours. No study has examined the minimum, and hence likely more behaviourally sustainable, improvements across all three SPAN behaviours required for measurable improvements in health outcomes.

The first aim of this study was to examine the combined associations of SPAN exposures with the risk of all-cause mortality in a large UK cohort using device-measured sleep, physical activity, and a comprehensive diet quality score for nutrition. Our second aim was to identify the minimum and optimal between-individual variations in sleep, physical activity and diet associated with clinically meaningful lower all-cause mortality risk.

## Methods

### Study population

We used data from the UK Biobank cohort study which recruited 502,629 adults aged 40–69 from 2006 to 2010 [25, 26]. Participants completed touchscreen questionnaires for sociodemographic information, lifestyle characteristics, and health status. All participants provided informed consent, and the ethical approval was completed by the UK National Health Service and National Research Ethics Service for the UK (No. 11/NW/0382).

**Fig. 1:**
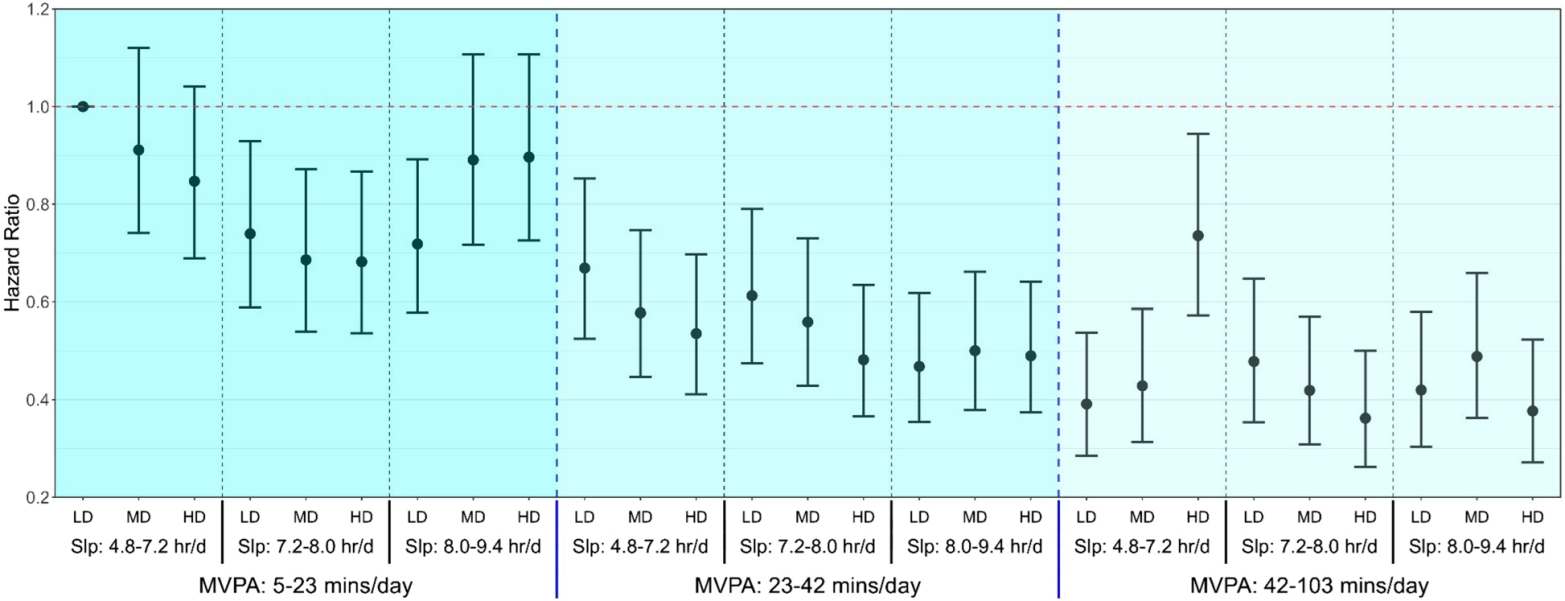
Multivariable-adjusted associations of combined Sleep, Physical Activity, and Nutrition with all-cause mortality risk. Legend: Model is adjusted for age, sex, ethnicity, smoking, education, Townsend deprivation index, alcohol, discretionary screen time (time spent watching TV or using the computer outside of work), light intensity physical activity, medication (blood pressure, insulin, and cholesterol), previous diagnosis of major CVD (defined as disease of the circulatory system, arteries, and lymph, excluding hypertension), previous diagnosis of cancer, and familial history of CVD and cancer (*n* = 59,078; events = 2,458). Sleep (hours/day), physical activity (moderate to vigorous intensity (MVPA) minutes/day), and nutrition (Dietary Quality Score (DQS)) were included in the model as a joint term. The specific ranges for each exposure included sleep duration as 4.8-7.2 hours/day (low), 7·2-8.0 hours/day (medium), and 8.0-9.4 hours/day (high); MVPA measurements as 5-23 minutes/day (low), 23-42 minutes/day (medium), and 42-103 minutes/day (high); and diet quality using the DQS as 32.5-50.0 (low), 50.0-57.5 (medium), and 57.5-72.5 (high). The lowest tertiles for all three exposures (sleep, MVPA and DQS) was considered the reference group. Dashed blue lines separate tertiles MVPA and dashed black lines separate tertiles of sleep. Sleep (Slp); Low Diet Quality (LD); Medium Diet Quality (MD); High Diet Quality (HD)

During 2013 to 2015, a subgroup of 103,684 participants were mailed and wore wrist-worn accelerometers (Axivity AX3, York, UK) on their dominant wrist for 7 days. Participants were only included in the present study if they had wrist-worn accelerometry data collected with a sufficient wear time of at least three days (> 16 h/day) with one of the days being a weekend day [27–31]. Participants were also excluded from the primary analysis if no sleep data was recorded, the accelerometer was poorly calibrated, or a faulty accelerometer was distributed. We also excluded participants who reported they were unable to walk or with incomplete covariate information (Fig. 1).

### Sleep, physical activity, and nutrition

Sleep and physical activity information was derived from wrist accelerometry data. All accelerometers were calibrated and initialized to 100 Hz, and participants were instructed to wear the device on their dominant wrist for 7 consecutive days. Sleep was defined as the average daily duration of sleep (hours/day) as calculated using a validated algorithm based on relative changes in wrist tilt angle between successive 5-s windows [32, 33]. Physical activity was defined as daily minutes of moderate to vigorous physical activity (MVPA) estimated using a validated two-stage machine learning scheme [28, 31, 34, 35]. Further detail regarding the classification system is described in the Additional Files 1: Methods.

Dietary data was derived using a previously validated twenty-nine item self-report food-frequency questionnaire (FFQ) [36–38], collected at recruitment (2006–2010) to determine the frequency of commonly consumed foods and food groups over the past 12-month period. From the recorded FFQ dietary information, we calculated a previously established diet quality score (DQS) which emphasizes higher intake of vegetables, fruits, fish, dairy, whole grains, and vegetable oils and lower intake of refined grains, processed meats, unprocessed red meats, and sugar-sweetened beverages (Additional Files 1: Supplementary Table 1) [39–42]. This score rates the intake of each food/beverage category on a scale from 0 (unhealthiest) to 10 (healthiest) for a total of 100 points, where higher values equate to a higher diet quality.

### Outcome ascertainment

Participants were followed through November 30th, 2022, with all-cause mortality information including follow-up duration and mortality events retrieved via the data linkage program from the National Health Service Central Register and National Records of Scotland. To avoid potential reverse causation, we excluded all participants with a mortality event in the first year of follow-up to omit individuals who may have altered lifestyle behaviours due to declining health status [27, 29].

### Statistical analyses

We estimated the associations between SPAN lifestyle characteristics and all-cause mortality using Cox proportional hazard regression models. To minimize the influence of sparse data and outliers, the values below the 2.5 percentile and above the 97.5 percentile for all SPAN exposures were Winsorized in this analysis [43].

We first estimated relative dose–response plots for each SPAN exposures to explore the minimum combined dose of SPAN exposures associated with a clinically meaningful reduction in all-cause mortality risk. As per previous work [44], we defined clinically meaningful lower all-cause mortality risk as a 10% reduction in risk or the nearest predicted integer. To identify the minimum variation associated with a clinically meaningful reduction in mortality risk, we created a combination matrix of all three exposures and selected the combinations associated with this level of risk reduction. For a broader list of clinically meaningful combinations, we plotted a heatmap correlogram that uses the combined 5th percentile of all three exposures (sleep (5.5 h/day), physical activity (7.3 min/day), and nutrition (36.9 DQS)) as reference while providing a corresponding hazard ratio for incremental amounts of each exposure as independent terms (i.e., 10th, 30th, 50th, 70th, and 90th percentile rounded to nearest 5th integer). We also explored associations of individual SPAN behaviours with all-cause mortality risk via dose–response plots using an ‘unhealthy’ reference (i.e., 5th percentile for each exposure) and a ‘healthy’ reference (i.e., median value for the two behaviours not shown: sleep (7.6 h per day), physical activity (31.2 min/day MVPA), and nutrition (54.3 DQS). In addition, we also created dose–response plots using guideline-oriented references for the individual SPAN behaviours. This included the minimum recommendation sleep for adults (7 h/day) [45], 150 min per week of MVPA for physical activity (approximately 20 min/day) [2], and given the absence of guidelines for DQS score, we used approximate percentile comparable to the other behaviours (25th percentile: 47.5 DQS).

To examine the optimal SPAN combination, participants then were grouped by SPAN combined SPAN exposure tertiles (i.e., low, moderate, and high) which equated to a joint exposure of 27 separate groups for all three SPAN behaviours. The sample size and number of all-cause mortality events for each group are detailed in Additional Files 1: Supplementary Table 2. The specific ranges for each exposure included sleep duration as 4.8–7.2 h/day (low), 7.2–8.0 h/day (medium), and 8.0–9.4 h/day (high); MVPA measurements as 5–23 min/day (low), 23–42 min/day (medium), and 42–103 min/day (high); and diet quality using the DQS as 32.5–50.0 (low), 50.0–57.5 (medium), and 57.5–72.5 (high). In these models, we included a joint SPAN exposure comprising 27 (mutually exclusive) categories reflecting all tertile combinations of the three individual exposures, with the lowest combined tertile serving as the referent category.

Models were adjusted for age, sex, ethnicity, smoking, education, Townsend deprivation index, alcohol, self-reported discretionary screen time, light intensity physical activity, medication (blood pressure, insulin, and cholesterol), previous diagnosis of major CVD, previous diagnosis of cancer, and familial history of CVD and cancer (Additional Files 1: Supplementary Table 3 provides full covariate definitions). We adjusted for self-reported discretionary rather than device measured sedentary behaviour to avoid a high degree of multicollinearity across the time-based movement behaviours [46, 47]. There was no evidence of problematic multicollinearity in the primary models or when adjusting for device measured sedentary behaviour (Additional Files 1: Supplementary Table 4–5) [48].

To test for interactive and synergistic effects, we calculated the relative excess risk due to interaction (RERI), attributable proportion due to interaction (AP), and the synergistic effects index (S) [49]. These tests provide insight into the contribution of synergistic interactions between exposures where an RERI or AP of 0 and an S value of 1 denote no interaction effect. All Cox models satisfied proportional hazards assumptions using Schoenfeld residuals.

### Sensitivity analyses

We adjusted a sensitivity model for other sleep characteristics from a previously established sleep score reflective of sleep quality including self-reported insomnia, snoring, chronotype (morning/evening person), and daytime sleepiness [23]. We also employed an alternative marker of dietary patterns by repeating main analyses using the proportion of dietary ultra-processed food (Additional Files 1: Supplementary Table 6) [36, 50–52]. We further adjusted the DQS model for total energy intake and excluded participants with sex-specific implausible ranges of total energy intake [53]. We repeated the main analyses after excluding participants who self-reported poor health, current smokers, those in the top 20th percentile of the frailty index, and those with an underweight BMI (< 18.5). In separate sensitivity models we also adjusted for BMI [54, 55]. We repeated the main analyses excluding those with prevalent CVD or cancer at baseline. We also completed a sensitivity analysis excluding rather than Winsorizing potential sparse data or outliers. We also provide a sensitivity analysis where we adjusted for device measured sedentary behaviour rather than self-reported discretionary screen time.

We undertook all statistical analyses and visualizations using the survival, rms, ggplot2 packages of R (version 4.3.1). We followed the Strengthening the Reporting of Observational Studies in Epidemiology (STROBE) guidelines (Additional Files 1: Supplementary Table 7).

## Results

### Sample

The final analytic sample included 59,078 participants (Median age [IQR]: 64.0 [7.8] years; 45.4% male) and 2,458 all-cause mortality events (Table 1). After excluding mortality events in the first 12 months of follow-up, the median follow-up period was 8.1 [7.5–8.6] years.

**Table 1:** Participant characteristics

	**Overall**	**Sleep**			**Physical activity**			**Nutrition**		
		**Low**	**Moderate**	**High**	**Low**	**Moderate**	**High**	**Low**	**Moderate**	**High**
**Sample**	59,078	19,693	19,692	19,693	19,693	19,692	19,693	20,918	19,464	18,696
**Events, n**	2,458	980	698	780	1,276	693	489	856	783	819
**Follow up, years (median [IQR])**	8.06 [7.49, 8.59]	8.05 [7.48, 8.56]	8.07 [7.51, 8.59]	8.05 [7.51, 8.59]	8.05 [7.46, 8.59]	8.06 [7.52, 8.59]	8.07 [7.52, 8.56]	8.06 [7.50, 8.59]	8.06 [7.50, 8.59]	8.05 [7.49, 8.56]
**Age, years (median [IQR])**	64.00 [57.00, 69.00]	64.00 [57.00, 69.00]	63.00 [56.00, 68.00]	64.00 [57.00, 69.00]	66.00 [60.00, 70.00]	64.00 [57.00, 69.00]	62.00 [55.00, 67.00]	62.00 [55.00, 68.00]	64.00 [57.00, 69.00]	65.00 [59.00, 70.00]
**Male, n (%)**	26,810 (45.4%)	10,068 (51.1%)	10,822 (55.0%)	11,378 (57.8%)	8,445 (42.9%)	9,002 (45.7%)	9,363 (47.5%)	10,582 (50.6%)	8,115 (41.7%)	8,113 (43.4%)
**Sleep, hours/day (median [IQR])**	7.62 [6.89, 8.26]	6.52 [5.92, 6.89]	7.62 [7.41, 7.83]	8.52 [8.26, 8.89]	7.60 [6.78, 8.30]	7.65 [6.92, 8.27]	7.62 [6.94, 8.22]	7.61 [6.86, 8.26]	7.62 [6.90, 8.25]	7.64 [6.90, 8.27]
**Moderate to vigorous physical activity, mins/day (median [IQR])**	31.26 [18.52, 49.09]	30.17 [17.52, 48.19]	33.05 [19.79, 51.31]	30.60 [18.24, 47.79]	14.31 [9.64, 18.52]	31.26 [26.76, 36.26]	58.74 [49.09, 74.91]	30.57 [18.24, 48.31]	31.67 [18.76, 49.43]	31.60 [18.59, 49.64]
**Diet quality score (median [IQR])**	54.29 [47.50, 60.00]	53.93 [47.50, 60.00]	54.01 [47.50, 60.00]	54.64 [47.50, 60.00]	53.93 [47.50, 60.00]	54.29 [47.50, 60.00]	54.29 [47.50, 60.00]	45.00 [40.00, 47.50]	55.00 [52.50, 56.79]	62.50 [60.00, 67.50]
**Light physical activity, mins/day (median [IQR])**	102.84 [70.24, 158.12]	106.72 [72.26, 162.46]	105.17 [72.00, 162.25]	96.81 [66.72, 149.53]	83.96 [59.79, 118.6]	107.43 [73.38, 157.72]	130.86 [81.72, 195.7]	100.81 [69.03, 155.55]	103.91 [71.26, 158.90]	103.98 [70.53, 159.82]
**Discretionary screen time, hours/day (median [IQR])**	3.50 [2.50, 5.00]	4.00 [2.50, 5.00]	3.50 [2.50, 5.00]	3.50 [2.50, 5.00]	4.00 [3.00, 5.00]	3.50 [2.50, 5.00]	3.00 [2.00, 4.50]	4.00 [2.50, 5.00]	3.50 [2.50, 5.00]	3.50 [2.50, 5.00]
**Smoking history, n (%)**	-	-	-	-	-	-	-	-	-	-
**Never**	34,024 (57.6%)	10,910 (554%)	11,508 (58.4%)	11,606 (58.9%)	10,781 (54.7%)	11,462 (58.2%)	11,781 (59.8%)	11,928 (57.0%)	11,385 (58.5%)	10,711 (57.3%)
**Former**	21,361 (36.2%)	7,320 (37.2%)	7,026 (35.7%)	7,015 (35.6%)	7,390 (37.5%)	7,076 (35.9%)	6,895 (35.0%)	7,236 (34.6%)	7,039 (36.2%)	7,086 (37.9%)
**Current**	3,693 (6.3%)	1,463 (7.4%)	1,158 (5.9%)	1,072 (5.4%)	1,522 (7.7%)	1,154 (5.9%)	1,017 (5.2%)	1,754 (8.4%)	1,040 (5.3%)	899 (4.8%)
**Alcohol consumption, units/week**^**b**^** (median [IQR])**	9.75 [1.70, 19.50]	9·52 [0.97, 19.50]	9.75 [2.44, 19.50]	9.75 [2.27, 19.57]	8·77 [0.62, 18.84]	9.75 [2.27, 19.50]	10.71 [3.20, 20.52]	9.75 [1.22, 20.13]	9.75 [2.44, 19.50]	9.75 [1.94, 19.50]
**Total energy intake, kcal/day (median [IQR])**^**a**^	2,018 [1,651, 2,443]	2,056 [1,676, 2,500]	2,026 [1,662, 2,441]	1,975 [1,617, 2,392]	1,965 [1,607, 2,382]	2,011 [1,649, 2,435]	2,075 [1,699, 2,511]	2,045 [1,677, 2,490]	2,017 [1,652, 2,433]	1,989 [1,620, 2,413]
**Townsend Deprivation Index, (median [IQR]])**	−2.49 [−3.84, −0.26]	−2.33 [−3.76, 0.13]	−2.50 [−3.83, −0.34]	−2.64 [−3.91, −0.59]	−2.41 [−3.78, −0.12]	−2.54 [−3.88, −0.34]	−2.52 [−3.85, −0.31]	−2.44 [−3.81, −0.09]	−2.52 [−3.87, −0.40]	−2.52 [−3.84, −0.33]
**Education, n (%)**	-	-	-	-	-	-	-	-	-	-
**College/University**	7,707 (13.0%)	2,615 (13.3%)	2,533 (12.9%)	2,559 (13.0%)	2,523 (12.8%)	2,605 (13.2%)	2,579 (13.1%)	2,740 (13.1%)	2,636 (13.5%)	2,331 (12.5%)
**A/AS**	25,619 (43.4%)	8,642 (43.9%)	8,882 (45.1%)	8,095 (41.1%)	8,095 (41.1%)	8,725 (44.3%)	8,799 (44.7%)	8,574 (41.0%)	8,780 (45.1%)	8,265 (44.2%)
**O levels**	3,262 (5.5%)	1,090 (5.5%)	1,065 (5.4%)	1,107 (5.6%)	1,145 (5.8%)	1,097 (5.6%)	1,020 (5.2%)	1,264 (6.0%)	989 (5.1%)	1,009 (5.4%)
**CSE**	12,028 (20.4%)	3,897 (19.8%)	3,923 (19.9%)	4,208 (21.4%)	4,104 (20.8%)	3,982 (20.2%)	3,942 (20.0%)	4,422 (21.1%)	3,896 (20.0%)	3,710 (19.8%)
**NVQ/HND/HNC**	2,216 (3.8%)	716 (3.6%)	714 (3.6%)	786 (4.0%)	642 (3.3%)	685 (3.5%)	889 (4.5%)	943 (4.5%)	669 (3.4%)	604 (3.2%)
**Other**	8,246 (14.0%)	2,733 (13.9%)	2,575 (13.1%)	2,938 (14.9%)	3,184 (16.2%)	2,598 (13.2%)	2,464 (12.5%)	2,975 (14.2%)	2,494 (12.8%)	2,777 (14.9%)
**Parental history of CVD, n (%)**	33,120 (56.1%)	11,074 (56.2%)	10,976 (55.7%)	11,070 (56.2%)	11,510 (58.4%)	11,004 (55.9%)	10,606 (53.9%)	11,144 (53.3%)	11,050 (56.8%)	10,926 (58.4%)
**Parental history of cancer, n (%)**	18,549 (31.4%)	6,177 (314%)	6,150 (31.2%)	6,222 (31.6%)	6,162 (31.3%)	6,252 (31.7%)	6,135 (31.2%)	6,509 (31.1%)	6,167 (31.7%)	5,873 (31.4%)
**Previous CVD, n (%)**	5,678 (9.6%)	2,132 (10.8%)	1,754 (8.9%)	1,792 (9.1%)	2,668 (13.5%)	1,732 (8.8%)	1,278 (6.5%)	1,971 (9.4%)	1,796 (9.2%)	1,911 (10.2%)
**Previous Cancer, n (%)**	5,145 (8.7%)	1,676 (8.5%)	1,672 (8.5%)	1,797 (9.1%)	2,036 (10.3%)	1,715 (8.7%)	1,394 (7.1%)	1,709 (8.2%)	1,737 (8.9%)	1,699 (9.1%)
**Ethnicity, n (%)**	-	-	-	-	-	-	-	-	-	-
**Other**	3,565 (6.0%)	1,552 (7.9%)	1,115 (5.7%)	898 (4.6%)	1,160 (5.9%)	1,162 (5.9%)	1,243 (6.3%)	1,257 (6.0%)	1,186 (6.1%)	1,122 (6.0%)
**White**	55,513 (94.0%)	18,141 (92.1%)	18,577 (94.3%)	18,795 (95.4%)	18,533 (94.1%)	18,530 (94.1%)	18,450 (93.7%)	19,661 (94.0%)	18,278 (93.9%)	17,574 (94.0%)
**Medication Use, n (%)**	-	-	-	-	-	-	-	-	-	-
**Cholesterol**	9,130 (15.5%)	3,426 (17.4%)	2,756 (14.0%)	2,948 (15.0%)	4,200 (21.3%)	2,870 (14.6%)	2,060 (10.5%)	2,835 (13.6%)	2,855 (14.7%)	3,440 (18.4%)
**Blood pressure**	5,384 (9.1%)	1,997 (10.1%)	1,680 (8.5%)	1,707 (8.7%)	2,287 (11.6%)	1,723 (8.7%)	1,374 (7.0%)	1,782 (8.5%)	1,749 (9.0%)	1,853 (9.9%)
**Insulin**	72 (0.1%)	21 (0.1%)	29 (0.1%)	22 (0.1%)	22 (0.1%)	27 (0.1%)	23 (0.1%)	23 (0.1%)	18 (0.1%)	31 (0.2%)
**Frailty Index > 3, n (%)**	196 (0.3%)	95 (0.5%)	51 (0.3%)	50 (0.3%)	142 (0.8%)	24 (0.1%)	30 (0.2%)	91 (0.5%)	53 (0.3%)	52 (0.3%)
**Self-rated health, n (%)**	-	-	-	-	-	-	-	-	-	-
**Excellent**	12,827 (21.8%)	3,930 (20.0%)	4,534 (23.1%)	4,363 (22.2%)	3,148 (16.0%)	4,382 (22.3%)	5,297 (26.9%)	4,138 (19.8%)	4,322 (22.2%)	4,367 (23.4%)
**Good**	35,514 (60.2%)	11,533 (58.7%)	11,970 (60.9%)	12,011 (61.1%)	11,583 (58.9%)	12,099 (61.5%)	11,832 (60.2%)	12,421 (59.5%)	11,897 (61.2%)	11,196 (60.0%)
**Fair**	9,175 (15.6%)	3,563 (18.1%)	2,774 (14.1%)	2,838 (14.4%)	4,083 (20.8%)	2,803 (14.3%)	2,289 (11.6%)	3,654 (17.5%)	2,814 (14.5%)	2,707 (14.5%)
**Poor**	1,458 (2.5%)	634 (3.2%)	378 (1.9%)	446 (2.3%)	844 (4.3%)	374 (1.9%)	240 (1.2%)	660 (3.2%)	394 (2.0%)	404 (2.2%)
**Body mass index, kg/m**^**2**^** (median [IQR])**	26.10 [23.60, 29.00]	26.70 [24.10, 29.90]	25.80 [23.50, 28.70]	25.70 [23.30, 28.50]	27.20 [24.50, 30.50]	26.00 [23.60, 28.80]	25.20 [23.00, 27.70]	26.40 [23.90, 29.40]	26.00 [23.60, 28.90]	25.80 [23.40, 28.70]

^a^Energy intake was calculated for a subsample of those with 24-h dietary recall (*n* = 43,695)

^b^Units/week (1 unit = 8 g of pure ethanol)

### Dose–response associations of individual SPAN behaviours with mortality

Compared to the 5th percentile, an additional 2 min/day of MVPA was associated with approximately 10% lower mortality risk (HR: 0.89; 95% CI: 0.88, 0.91; Additional Files 1: Supplementary Fig. 2). An additional 24 min/day of sleep was associated with approximately 10% lower risk for all-cause mortality (HR: 0.91; 95% CI: 0.88, 0.94). DQS revealed a subtle but not statistically significant dose–response relationship with mortality.

At the median level of SPAN behaviours (Additional Files 1: Supplementary Fig. 2), an additional 3 min/day of MVPA (HR: 0.71; 95% CI: 0.64, 0.79) or an additional 36 min of sleep (HR: 0.63; 95% CI: 0.64, 0.79) was associated with a 10% lower risk for all-cause mortality. DQS was significantly associated with all-cause mortality but did not reach a clinically meaningful lower risk of all-cause mortality in isolation.

When compared to the guideline-based reference for sleep (7 h/day), physical activity (20 min/day of MVPA), and nutrition (47.5 DQS), an additional 3.5 min/day of MVPA (HR: 0.89; 95% CI: 0.88, 0.91) was associated with a 10% lower all-cause mortality risk (Additional Files 1: Supplementary Fig. 3).

### Combined SPAN associations and all-cause mortality risk

The absolute risk as was highest in the low sleep, low MVPA, and low DQS tertiles (49.76 per 10,000 person years (PY); 95% CI: 42.09, 58.83; Additional Files 1: Supplementary Fig. 4) and lowest in the high MVPA (42–103 min/day), moderate sleep (7.2–8.0 h/day), and high DQS (57.5–72.5; Absolute Risk: 17.95 per 10,000 PY; 95% CI: 13.25, 24.31).

MVPA contributed most to the gradient in all-cause mortality risk followed by sleep duration and DQS (Fig. 1). Compared to the referent lowest combined tertile for all three exposures, low MVPA, moderate sleep and high DQS corresponded to a 32% lower risk for all-cause mortality (HR: 0.68; 95% CI: 0.54, 0.87). Comparatively, a combination of moderate MVPA, moderate sleep, and high DQS was associated with 52% lower mortality risk (HR: 0.48; 95% CI: 0.37, 0.63). The highest risk reduction was associated with high MVPA, moderate sleep, and high DQS which corresponded to a 64% lower risk for all-cause mortality (HR: 0.36; 95% CI: 0.26, 0.50). There was evidence for a synergistic interaction between the three behaviours and all-cause mortality, as indicated by the RERI (0.06; 95% CI: 0.004, 0.13), AP (11.7%; 95% CI: 1–39%), and S (0.89; 95% CI: 0.84, 0.97; Additional Files 1: Supplementary Table 8).

### Minimal variations across the three SPAN behaviours and all-cause mortality risk

**Fig. 2:**
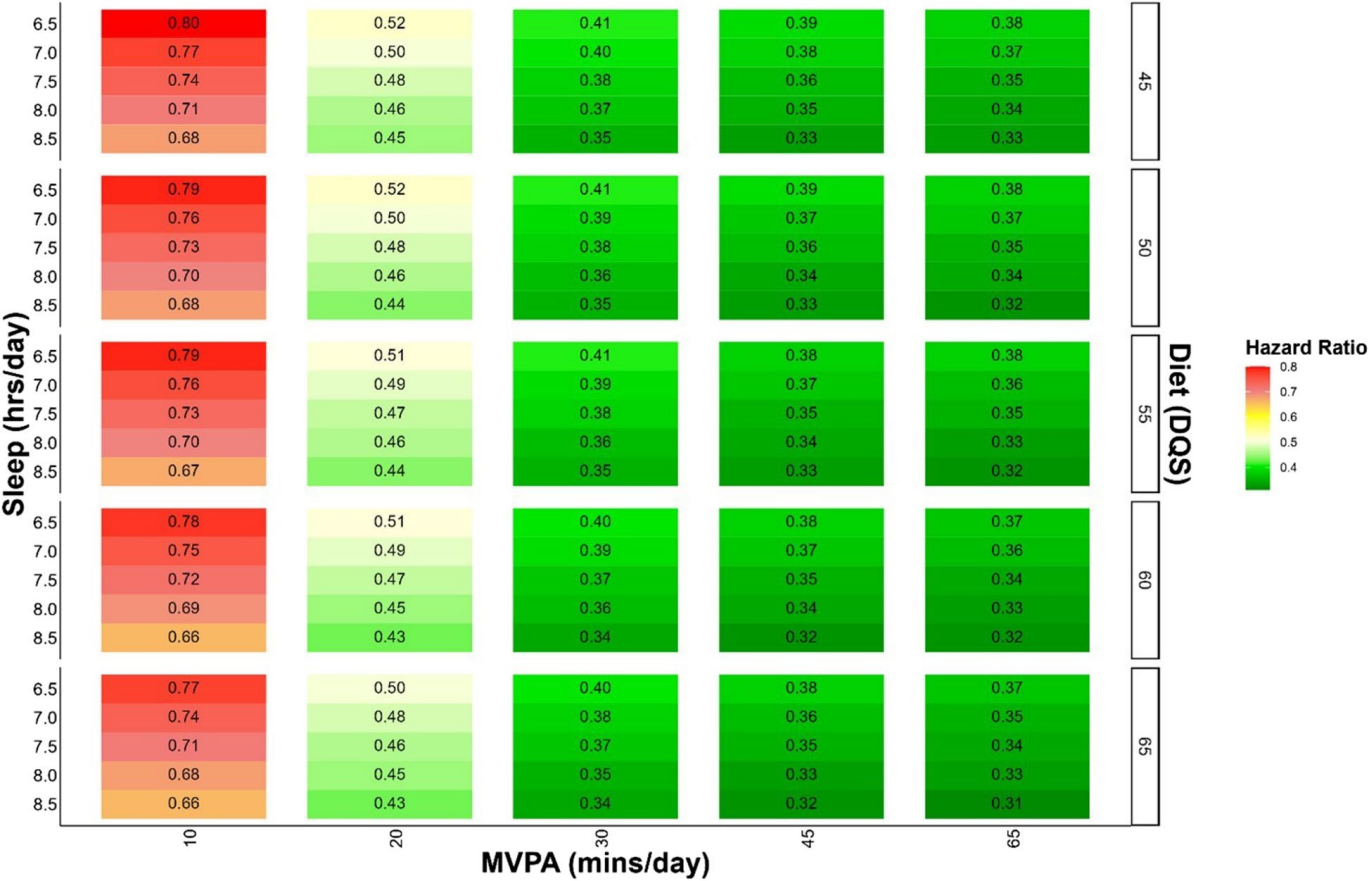
Multivariable adjusted all-cause mortality risk associated with concurrent variations in Sleep, Physical Activity, and Nutrition. Legend: The correlogram displays changes in sleep (hours/day), physical activity (moderate to vigorous intensity (MVPA) minutes/day), and nutrition (Dietary Quality Score (DQS)) and corresponding mortality risk with the reference being the 5^th^ percentile of sleep (5.5 hours/day), physical activity (7.3 minutes/day), and nutrition (36.9 DQS). Sleep, physical activity, and nutrition are included as independent terms in the model to allow for more granular predictions. Each square on the grid represents the hazard ratio for all-cause mortality associated with a combination of behaviours, as defined by the x-axis (physical activity), y-axis (sleep), and z-axis (nutrition). The colour corresponds to the hazard ratio where red indicates a higher risk of all-cause mortality and green indicates a lower risk of all-cause mortality. Model is adjusted for age, sex, ethnicity, smoking, education, Townsend deprivation index, alcohol, discretionary screen time (time spent watching TV or using the computer outside of work), light intensity physical activity, medication (blood pressure, insulin, and cholesterol), previous diagnosis of major CVD (defined as disease of the circulatory system, arteries, and lymph, excluding hypertension), previous diagnosis of cancer, and familial history of CVD and cancer (*n* = 59,078; events = 2,458)

Table 2 presents the combined doses of sleep, physical activity, and nutrition associated with different levels of all-cause mortality risk (10–70% range). The first rows indicate the minimal differences in the combined SPAN behaviours associated with clinically relevant lower risk for mortality. For example, compared to the combined SPAN reference point (5th percentile of sleep (5.5 h/day), physical activity (7.3 min/day), and nutrition (36.9 DQS)), the combination of an additional 15 min/day of sleep, 1.6 min/day MVPA, and 5 points DQS was associated with a 10% lower risk of all-cause mortality (HR: 0.90; 95% CI: 0.88, 0.93). The combination of an additional 75 min/day of sleep, 12.5 min/day MVPA, and 25 DQS points corresponds to a 50% lower risk of all-cause mortality (HR: 0.50; 95% CI: 0.44, 0.58). The optimal concurrent variation included an additional 200 min/day of sleep, 58.8 min/day of MVPA, and 35 DQS points corresponding to 70% lower all-cause mortality risk (HR: 0.30; 95% CI: 0.25, 0.38). For context, an additional 5 DQS points corresponds to increasing cooked vegetable consumption by 1/3 cup per day or increasing fresh fruit consumption by 1.5 pieces per day. The different combinations of SPAN exposures corresponding to all-cause mortality risk are graphically illustrated as heat map correlograms in Additional Files 1: Supplementary Fig. 5 (absolute risk) and Fig. 2 (hazard ratios).

**Table 2:** Minimum concurrent variations in Sleep, Physical Activity, and Nutrition associated with clinically relevant increments of all-cause mortality risk compared to 5th percentile of SPAN exposures

**All-cause mortality risk**^**c**^	**Additional Sleep** (min/day)	**Additional Moderate to Vigorous Physical Activity** (min/day)	**Additional Nutrition** (DQS points)	**Additional Sleep**^**a**^ (min/day)	**Additional Moderate to Vigorous Physical Activity**^**a**^ (min/day)	**Additional Nutrition**^**a**^ (DQS points)
	**COMBINED SPAN**			**INDIVIDUAL SPAN**		
10% lower risk (HR: 0·90; 95% CI: 0·88, 0·93)	15	1.6	5	24	2	17^b^
20% lower risk (HR: 0·80; 95% CI: 0·76, 0·84)	30	3.6	10	54	5	-
30% lower risk (HR: 0·70; 95% CI: 0·65, 0·76)	45	5.9	15	102	8	-
40% lower risk (HR: 0·60; 95% CI: 0·54, 0·67)	60	8.7	20	114	12	-
50% lower risk (HR: 0·50; 95% CI: 0·44, 0·58)	75	12.5	25	-	18	-
60% lower risk (HR: 0·40; 95% CI: 0·34, 0·48)	90	19.5	30	-	77	-
70% lower risk (HR: 0·30; 95% CI: 0·25, 0·38)	200	58.8	35	-	-	-

Displays the minimum concurrent combinations of sleep, physical, activity and nutrition associated with a clinically meaningful lower all-cause mortality risk compared to individual SPAN exposures. The combined columns show the SPAN combinations and corresponding mortality risk compared to the 5th percentile of sleep (5.5 h/day), moderate to vigorous physical activity (7.3 min/day), and nutrition (36.9 DQS) in increments of 10%. For comparison, the dose needed for individual SPAN exposures is shown on the right. Empty cells denote that the individual SPAN exposure could not achieve that level of risk reduction in isolation. All models were adjusted for age, sex, ethnicity, smoking, education, Townsend deprivation index, alcohol, discretionary screen time (time spent watching TV or using the computer outside of work), light intensity physical activity, medication (blood pressure, insulin, and cholesterol), previous diagnosis of major CVD (defined as disease of the circulatory system, arteries, and lymph, excluding hypertension), previous diagnosis of cancer, and familial history of CVD and cancer. Moderate to vigorous physical activity (MVPA); Diet quality score (DQS); Hazard ratio (HR)

^a^Each exposure was adjusted by the median value of the other two SPAN exposures

^b^Here, HR = 0·94. This is the lowest adjusted HR for DQS individually

^c^95% CI are based on for combined SPAN model

### Sensitivity analyses

Excluding participants with poor health (*n* = 51,164; events = 1,887), previous CVD or cancer (*n* = 49,786; events = 1,637), potentially sparse or outlier data (*n* = 48,670; events = 1,888), adjusting for device measured sedentary behaviour (*n* = 59,078; events = 2,458), adjusting for BMI (*n* = 58,363; events = 2,405), or adjusting for other sleep characteristics (*n* = 37,475; events = 1,506) did not materially influence the results (Additional Files 1: Supplementary Figs. 6–11). Categorising diet quality by the proportion of ultra-processed food intake (*n* = 41,936; events = 1,758; Additional Files 1: Supplementary Fig. 12) and adjustment for total energy intake (*n* = 42,990; events = 1,758; Additional Files 1: Supplementary Fig. 13) produced results consistent with the main findings. Some sensitivity analyses showed marginal differences in the optimal group, these were likely influenced by the reduced sample size and low event numbers.

## Discussion

In the first study of its kind, we estimated the minimal and optimal combined incremental variations across sleep, physical activity and nutrition that are associated with meaningful reductions in all-cause mortality risk. We utilized a novel 3-exposure joint association approach and data from the wearables study of the UK Biobank for sleep and physical activity measurements [27–29, 35]. The optimal combination of high MVPA, moderate sleep, and high DQS corresponded to a 64% lower risk for all-cause mortality compared to the reference group (lowest tertiles of all exposures). In practice, such a risk reduction would demand substantial and likely unrealistic improvements in any one behaviour (e.g., an extra > 78 min/day of MVPA). It was therefore particularly encouraging that our findings revealed that very modest combined doses of SPAN behaviours (e.g., an additional 15 min/day of sleep, 1.6 min/day MVPA, and 5 DQS points) were associated with a meaningful difference in all-cause mortality risk of 10%. Our findings support the existence of a synergistic association, as higher diet quality was only associated with lower mortality risk in combination with more optimal sleep and physical activity (Table 2), an assertion also supported by the results of the RERI tests. While an existing body of research highlights the pairwise effects of physical activity, nutrition, and sleep [15, 22, 23, 56], limited research exists that explores all three factors in combination, particularly the combined incremental benefits on mortality.

A previous 45 and Up Study [57] analysis examining the relationship between dichotomized (healthy-unhealthy) lifestyle behaviour risk factors (alcohol, smoking, physical inactivity, poor sleep, poor diet) and mortality risk in a group of 231,048 Australian participants showed that short sleep duration, physical inactivity, and poor diet had a 49% higher risk of all-cause mortality compared to the optimal behaviours referent group. Other work from the HUNT Study in Norway (*n* = 36,911) demonstrated that short sleep duration, physical inactivity and poor diet, combined with lack of social participation, led to an over two-fold increase in risk of all-cause mortality (HR: 2.26; 95% CI: 1.91–2.69) [58]. Importantly, none of the previous studies attempted to estimate the minimal dose of combined behaviours that are associated with practically important reductions in risk.

The individual associations of sleep, MVPA, and DQS, required a substantially higher dose to show associations of similar magnitude with mortality risk reduction compared to more optimal combined SPAN levels. For example, a 10% lower risk of mortality corresponded to 60% more sleep (24 min/day), 25% more MVPA (2 min/day) and diet alone, as measured using the chosen DQS, was unable to reach a 10% lower mortality risk in isolation. In comparison, when considering all SPAN behaviours in combination, a minimum dose of only 15 min/day for sleep, 1.6 min/day MVPA, and 5 DQS was sufficient to achieve this goal. For diet quality, this translates into making one of the relatively modest changes such as consuming an additional 1.5 servings of fresh fruit per day, increasing vegetable intake by 1/3 cup cooked vegetables per day, or adding one serving of fatty fish per week. An increase of 25 DQS points corresponds to multiple dietary changes such as consuming an extra 1/3 cup per day of cooked vegetables in addition to reducing refined grains intake by 1 serving per week, reducing processed meat by 1 serving per week, and eliminating consumption of sugar-sweetened beverages. This study emphasises that combined doses of SPAN behaviours act synergistically, whereby the collective behaviours were associated with a significantly stronger association with all-cause mortality risk than their individual components. Unlike the small body of previous empirical studies examining combined changes in lifestyle behaviours [59, 60], our study evaluates whether subtle theoretical changes in 3 interrelated behaviours yield clinically meaningful improvements in mortality risk. Assuming causal relationships, our findings suggest that studies focused on individual SPAN behaviours may be potentially missing a powerful and more behaviourally sustainable opportunity to improve health outcomes through subtle behaviour changes.

A key finding of this study is the unique synergies between these behaviours, likely driven by their behavioural interdependencies [13–18]. By examining SPAN as a combination of behaviours, this study harnesses these synergies to maximise health improvements and provide tangible public health guidance to assist individuals and practitioners in identifying the most feasible behavioural lifestyle changes. This is a crucial point for reducing the risk of noncommunicable disease and mortality considering the multiple barriers to major SPAN improvements such as the inability to find time or motivation for an extra 25–30 min of leisure time physical activity, limited cooking skills, or unavoidable disruptions to sleep patterns. If replicated by additional observational studies and behavioural trials, these findings expand options for holistic, combined lifestyle recommendations that can co-exist alongside traditional uni-behavioural advice.

### Strengths and limitations

We used a novel analytic approach estimating varying levels of multiple influential lifestyle behaviours and the corresponding association with all-cause mortality risk, allowing us to explore both the combined and relative associations of individual SPAN components. This is of particular importance considering the well-established co-dependencies between each of the SPAN exposures [15, 17, 19, 23, 24, 57]. The study is further strengthened due to the device-based measurement of both sleep and physical activity. By utilizing wearable devices for data collection, the study effectively mitigates the common pitfalls associated with self-reported data, such as under-reporting or over-reporting.

We acknowledge, however, that this latter strength is also a weakness when it comes to making comparisons between nutrition, which was measured by self-report in the UK Biobank, and sleep and physical activity, which were measured using wearable devices. This introduces some potential misalignment across SPAN exposures as device-measured physical activity has shown at least a threefold magnitude of associations with all-cause mortality [61]. Additionally, FFQ dietary data was collected 3–9 years earlier (2006–2010) than the wearable device measured sleep and physical activity (2013–2015). We conducted a range of additional analyses to strengthen the robustness of our findings, including the exclusion of those with a mortality event in the first year of follow up, self-rated poor health, high frailty index, and those with an underweight BMI. Despite these precautionary analyses, the possibility of reverse causation or residual confounding cannot be ruled out. Additionally, in the joint tertile-based analyses there was a relatively small number of mortality events in some of the healthiest SPAN categories, which may have affected the precision of the estimates in these groups. As this study is observational in nature and did not directly measure changes in behaviour, we cannot draw causal conclusions about behaviour-related risk reduction. Further longitudinal and interventional research is essential to confirm these findings and understand the sustainability of subtle SPAN behaviour modifications, induction times for meaningful health effects, and the influence of exposure length. Lastly, unhealthy lifestyle factors rarely occur in isolation, often forming broader deleterious behavioural patterns. This may have implications for other aspects of movement behaviour such as light physical activity, sedentary behaviour, and sleep regularity [62] all of which could have distinct synergistic relationships with mortality risk. Future studies with sufficient sample size and length of follow-up should explore different combinations of SPAN related behaviours, in addition to other lifestyle factors such as alcohol consumption, smoking, and other substance use.

## Conclusions

This study emphasises that combined SPAN behaviours act synergistically, whereby the collective behaviours were more strongly associated with all-cause mortality risk than the individual behaviours. We show that the optimal SPAN combination included high MVPA (42–103 min/day), moderate sleep (7.2–8.0 h/day), and high DQS (57.5–72.5), which corresponded to a 64% lower risk for all-cause mortality compared to the lowest combined tertile category for all three exposures. Our study also underscores the significance of incremental combined positive lifestyle behaviours. Compared to those with poor SPAN behaviours, very modest collective variations, such as a 15 min/day increase in sleep, less than 2 min/day higher MVPA, and higher diet quality equivalent to increasing daily intake of cooked vegetables by 1/3 cup or fruit by 1.5 servings, were associated with a 10% lower risk of all-cause mortality. If supported by future trials, our findings support the implementation of clinical and educational strategies and leveraging the potential of wearables and other digital technologies, aimed at encouraging concurrent small incremental improvements in sleep, physical activity, and nutrition.

## Supplementary Information

Additional file 1: Supplementary Methods. Wearable device-based Physical Activity and Sleep Classification. Supplementary Figure 1: Flow diagram of participants in the study. Supplementary Figure 2: Dose-response associations of each individual exposure (sleep duration, daily MVPA duration, and diet quality score) with all-cause mortality risk (*n* = 59,078; events = 2,458). Supplementary Figure 3: Dose-response associations of each individual exposure (sleep duration, daily MVPA duration, and diet quality score) with all-cause mortality risk using a guideline oriented reference (*n* = 59,078; events = 2,458). Supplementary Figure 4: Multivariable-adjusted associations of combined Sleep, Physical Activity, and Nutrition with absolute all-cause mortality risk per 10,000 person-years (*n* = 59,078; events = 2,458). Supplementary Figure 5: Absolute all-cause mortality risk associated with concurrent variations in sleep, MVPA, and dietary quality score (*n* = 59,078; events = 2,458). Supplementary Figure 6: Multivariable-adjusted associations of combined Sleep, Physical Activity, and Nutrition with all-cause mortality excluding poor health individuals (*n* = 51,164; events = 1,887). Supplementary Figure 7: Multivariable-adjusted associations of combined Sleep, Physical Activity, and Nutrition with all-cause mortality excluding individuals with baseline CVD or cancer (*n* = 49,786; events = 1,637). Supplementary Figure 8: Multivariable-adjusted associations of combined Sleep, Physical Activity, and Nutrition with all-cause mortality excluding individuals with potentially sparse or outlier data (*n *= 48,670; events = 1,888). Supplementary Figure 9: Multivariable-adjusted associations of combined Sleep, Physical Activity, and Nutrition with all-cause mortality adjusted for device measured sedentary behaviour (*n *= 59,078; events = 2,458). Supplementary Figure 10: Multivariable-adjusted associations of combined Sleep, Physical Activity, and Nutrition with all-cause mortality adjusted for BMI (*n* = 58,363; events = 2,405). Supplementary Figure 11: Multivariable-adjusted associations of combined Sleep, Physical Activity, and Nutrition with all-cause mortality adjusted for sleep characteristics (*n* = 37,475; events = 1,506). Supplementary Figure 12: Multivariable-adjusted associations of combined Sleep, Physical Activity, and Nutrition with all-cause mortality using the proportion of ultra-processed food (*n *= 41,936; events = 1,758). Supplementary Figure 13: Multivariable-adjusted associations of combined Sleep, Physical Activity, and Nutrition with all-cause mortality adjusted for total energy intake (*n* = 42,990; 1,758 events). Supplementary Table 1: Diet quality score index for food-frequency questionnaire dietary data.Supplementary Table 2: Sample size and all-cause mortality events for each Sleep, Physical Activity, and Nutrition category. Supplementary Table 3: Covariate definitions. Supplementary Table 4: Model variance inflation factors for combined SPAN behaviours. Supplementary Table 5: Model variance inflation factors for individual SPAN behaviours. Supplementary Table 6: NOVA classification of food groups for 24-hour dietary recall data. Supplementary Table 7: STROBE statement. Supplementary Table 8: Relative excess risk due to interaction.

## Data Availability

No datasets were generated or analysed during the current study.

## Abbreviations

SPAN: Sleep, physical activity, and nutrition
MVPA: Moderate to vigorous physical activity (mins/day)
FFQ: Food**-**frequency questionnaire
DQS: Diet quality score
RERI: Relative excess risk due to interaction
AP: Attributable proportion due to interaction
S: Synergistic effects index
