# Supplementary material for: Minimum and optimal combined variations in sleep, physical activity, and nutrition in relation to all-cause mortality risk
Source: BMC Med. 2025 Feb 26;23:111. doi: 10.1186/s12916-024-03833-x (PMC11863424; doi:10.1186/s12916-024-03833-x)
Supplement: Supplementary file 1 — Additional file 1: Supplementary Methods. Wearable device-based Physical Activity and Sleep Classification. Supplementary Figure 1: Flow diagram of participants in the study. Supplementary Figure 2: Dose-response associations of each individual exposure (sleep duration, daily MVPA duration, and diet quality score) with all-cause mortality risk (n = 59,078; events = 2,458). Supplementary Figure 3: Dose-response associations of each individual exposure (sleep duration, daily MVPA duration, and diet quality score) with all-cause mortality risk using a guideline oriented reference (n = 59,078; events = 2,458). Supplementary Figure 4: Multivariable-adjusted associations of combined Sleep, Physical Activity, and Nutrition with absolute all-cause mortality risk per 10,000 person-years (n = 59,078; events = 2,458). Supplementary Figure 5: Absolute all-cause mortality risk associated with concurrent variations in sleep, MVPA, and dietary quality score (n = 59,078; events = 2,458). Supplementary Figure 6: Multivariable-adjusted associations of combined Sleep, Physical Activity, and Nutrition with all-cause mortality excluding poor health individuals (n = 51,164; events = 1,887). Supplementary Figure 7: Multivariable-adjusted associations of combined Sleep, Physical Activity, and Nutrition with all-cause mortality excluding individuals with baseline CVD or cancer (n = 49,786; events = 1,637). Supplementary Figure 8: Multivariable-adjusted associations of combined Sleep, Physical Activity, and Nutrition with all-cause mortality excluding individuals with potentially sparse or outlier data (n = 48,670; events = 1,888). Supplementary Figure 9: Multivariable-adjusted associations of combined Sleep, Physical Activity, and Nutrition with all-cause mortality adjusted for device measured sedentary behaviour (n = 59,078; events = 2,458). Supplementary Figure 10: Multivariable-adjusted associations of combined Sleep, Physical Activity, and Nutrition with all-cause mortality adjusted for [file 12916_2024_3833_MOESM1_ESM.docx]

**ADDITIONAL FILES 1**

**Minimum and optimal combined variations in sleep, physical activity, and nutrition in relation to all-cause mortality risk**

**Table of Contents**

| **Page** | **Item** |
| --- | --- |
| **3** | **Supplementary Methods.** Wearable device-based Physical Activity and Sleep Classification |
| **4** | **Supplementary Figure 1**: Flow diagram of participants in the study |
| **5** | **Supplementary Figure 2:** Dose-response associations of each individual exposure (sleep duration, daily MVPA duration, and diet quality score) with all-cause mortality risk (n = 59,078; events = 2,458) |
| **6** | **Supplementary Figure 3:** Dose-response associations of each individual exposure (sleep duration, daily MVPA duration, and diet quality score) with all-cause mortality risk using a guideline oriented reference (n = 59,078; events = 2,458) |
| **7** | **Supplementary Figure 4:** Multivariable-adjusted associations of combined Sleep, Physical Activity, and Nutrition with absolute all-cause mortality risk per 10,000 person-years (n = 59,078; events = 2,458) |
| **8** | **Supplementary Figure 5:** Absolute all-cause mortality risk associated with concurrent variations in sleep, MVPA, and dietary quality score (n = 59,078; events = 2,458) |
| **9** | **Supplementary Figure 6:** Multivariable-adjusted associations of combined Sleep, Physical Activity, and Nutrition with all-cause mortality excluding poor health individuals (n = 51,164; events = 1,887) |
| **10** | **Supplementary Figure 7:** Multivariable-adjusted associations of combined Sleep, Physical Activity, and Nutrition with all-cause mortality excluding individuals with baseline CVD or cancer (n = 49,786; events = 1,637) |
| **11** | **Supplementary Figure 8:** Multivariable-adjusted associations of combined Sleep, Physical Activity, and Nutrition with all-cause mortality excluding individuals with potentially sparse or outlier data (n = 48,670; events = 1,888) |
| **12** | **Supplementary Figure 9:** Multivariable-adjusted associations of combined Sleep, Physical Activity, and Nutrition with all-cause mortality adjusted for device measured sedentary behaviour (n = 59,078; events = 2,458) |
| **13** | **Supplementary Figure 10:** Multivariable-adjusted associations of combined Sleep, Physical Activity, and Nutrition with all-cause mortality adjusted for BMI (n = 58,363; events = 2,405) |
| **14** | **Supplementary Figure 11:** Multivariable-adjusted associations of combined Sleep, Physical Activity, and Nutrition with all-cause mortality adjusted for sleep characteristics (n = 37,475; events = 1,506) |
| **15** | **Supplementary Figure 12:** Multivariable-adjusted associations of combined Sleep, Physical Activity, and Nutrition with all-cause mortality using the proportion of ultra-processed food (n = 41,936; events = 1,758) |
| **16** | **Supplementary Figure 13:** Multivariable-adjusted associations of combined Sleep, Physical Activity, and Nutrition with all-cause mortality adjusted for total energy intake (n = 42,990; 1,758 events) |
| **17** | **Supplementary Table 1:** Diet quality score index for food-frequency questionnaire dietary data |
| **18** | **Supplementary Table 2:** Sample size and all-cause mortality events for each Sleep, Physical Activity, and Nutrition category |
| **19** | **Supplementary Table 3:** Covariate definitions |
| **21** | **Supplementary Table 4:** Model variance inflation factors for combined SPAN behaviours |
| **22** | **Supplementary Table 5:** Model variance inflation factors for individual SPAN behaviours |
| **24** | **Supplementary Table 6:** NOVA classification of food groups for 24-hour dietary recall data |
| **25** | **Supplementary Table 7:** STROBE statement |
| **27** | **Supplementary Table 8:** Relative excess risk due to interaction |

**Supplementary Methods 1. Wearable device-based physical activity and sleep classification.**

Physical activity was defined as daily minutes of moderate to vigorous physical activity (MVPA) estimated using a validated two-stage machine learning scheme that first classifies each 10 second window (epoch) as sedentary (lying or sitting still), stationary plus (active sitting, standing still, active standing), walking, or running. Windows or epochs classified by the scheme as walking with normalized gravitational units ≥100 milli g and <400 milli g are considered moderate intensity physical activity[35]. Windows classified as walking with normalized gravitational units ≥ 400 milli g are considered vigorous intensity PA. All windows classified as running are considered vigorous intensity physical activity (≥ 6 METs)[29, 35, 36].

Sleep was defined as the average daily duration of sleep (hours/day) as calculated using a validated algorithm based on relative changes in wrist tilt angle between successive 5-second windows[33]. For each interval of 5 seconds, the average of the estimated wrist tilt angle was calculated and a rolling 5 minute median served as an input for the algorithm to identify sleep onset and sleep offset, and then time spent asleep within this timeframe[33, 34].

**
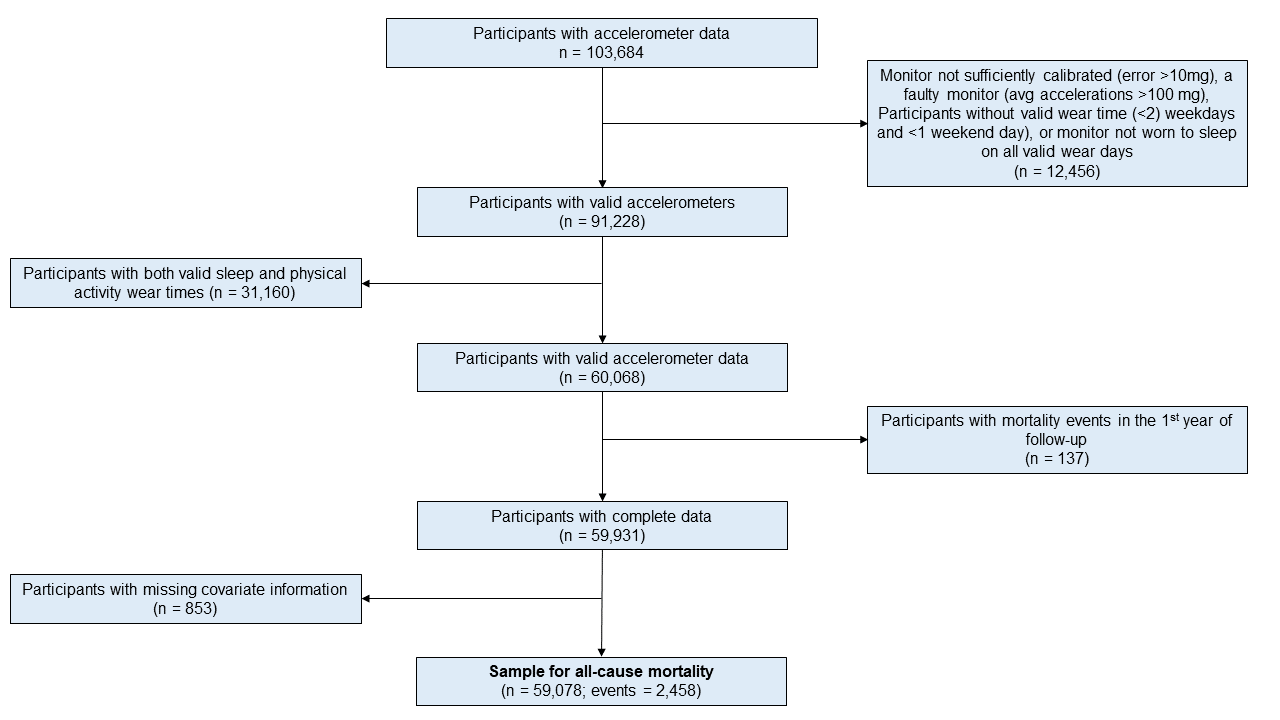
**

**Supplementary Figure 1. Participant flow chart**

**
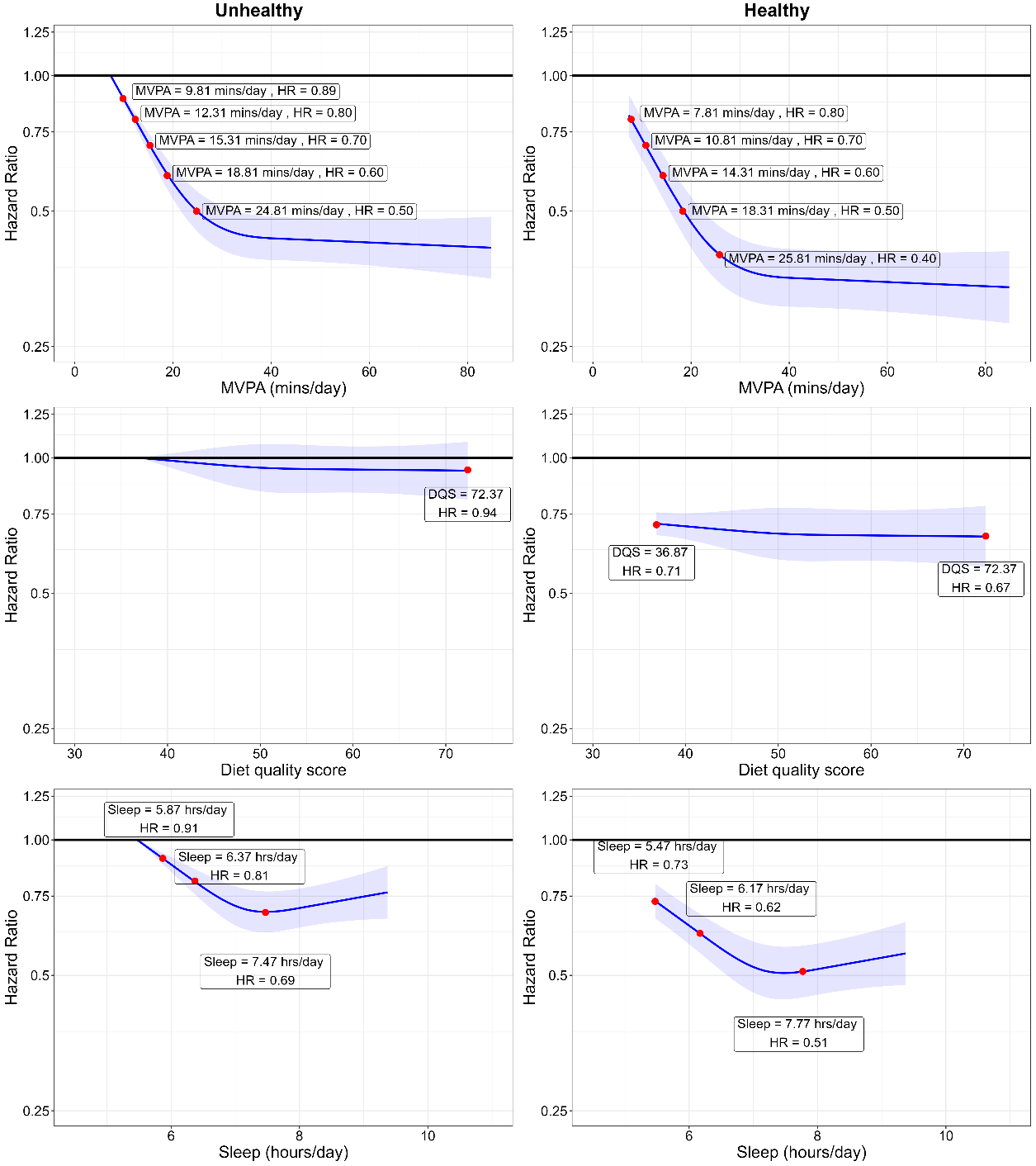
**

**Supplementary Figure 2: Dose-response associations of each individual exposure (sleep duration, daily MVPA duration, and diet quality score) with all-cause mortality risk (n = 59,078; events = 2,458)**

**Legend**: Dose-response plots for individual exposures are shown with an ‘unhealthy’ reference (i.e., 5^th^ percentile for each exposure) and a ‘healthy’ reference (i.e., median value for the two behaviours not shown: sleep (7.6 hours per day), physical activity (31.2 mins/day MVPA), and nutrition (54.3 DQS)). Red points denote risk reduction in increments of 10% (HR = 0.10) to the nearest value. Model is adjusted for age, sex, ethnicity, smoking, education, Townsend deprivation index, alcohol, discretionary screen time (time spent watching TV or using the computer outside of work), light intensity physical activity, medication (blood pressure, insulin, and cholesterol), previous diagnosis of major CVD (defined as disease of the circulatory system, arteries, and lymph, excluding hypertension), previous diagnosis of cancer, and familial history of CVD and cancer.


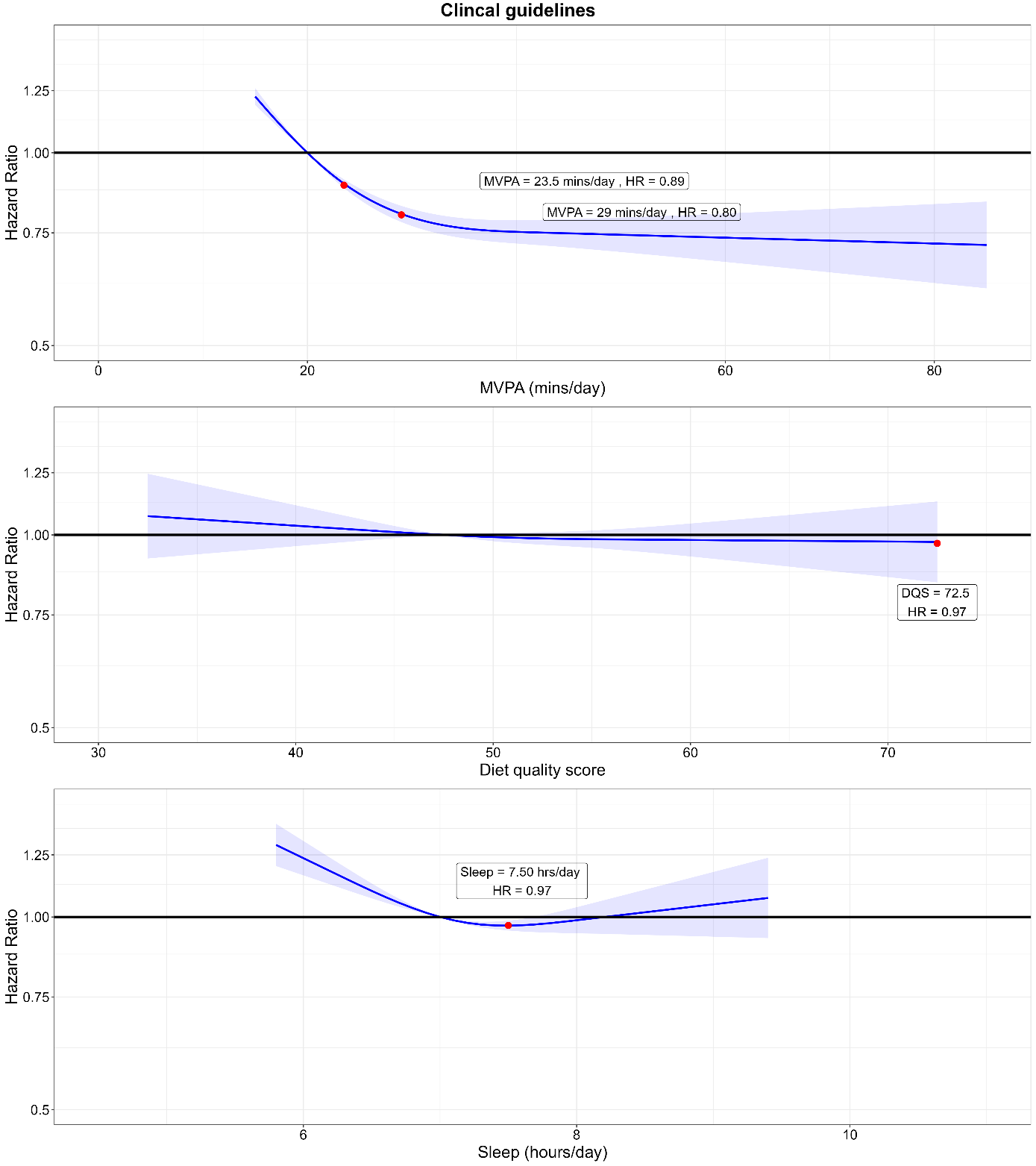


**Supplementary Figure 3: Dose-response associations of each individual exposure (sleep duration, daily MVPA duration, and diet quality score) with all-cause mortality risk using a guideline oriented reference (n = 59,078; events = 2,458)**

**Legends:** Dose-response plots for individual exposures use a guideline based[2, 46] reference for sleep (7 hours/day), physical activity (20 moderate to vigorous (MVPA) minutes/day), and nutrition (47.5 Diet Quality Score (DQS)). Red points denote risk reduction in increments of 10% (HR = 0.10) to the nearest value. Model is adjusted for age, sex, ethnicity, smoking, education, Townsend deprivation index, alcohol, discretionary screen time (time spent watching TV or using the computer outside of work), light intensity physical activity, medication (blood pressure, insulin, and cholesterol), previous diagnosis of major CVD (defined as disease of the circulatory system, arteries, and lymph, excluding hypertension), previous diagnosis of cancer, and familial history of CVD and cancer.


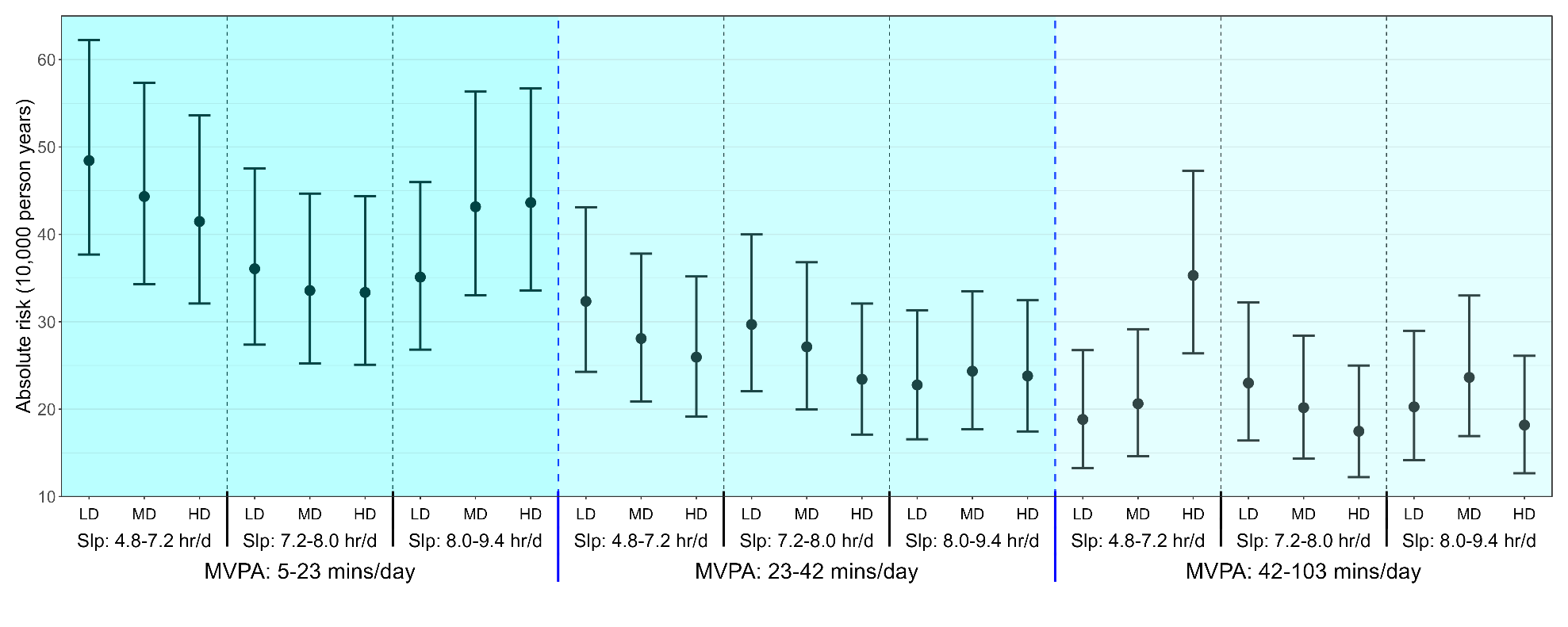


**Supplementary Figure 4: Multivariable-adjusted associations of combined Sleep, Physical Activity, and Nutrition with absolute all-cause mortality risk per 10,000 person-years (n = 59,078; events = 2,458)**

**Legend:** Model is adjusted for age, sex, ethnicity, smoking, education, Townsend deprivation index, alcohol, discretionary screen time (time spent watching TV or using the computer outside of work), light intensity physical activity, medication (blood pressure, insulin, and cholesterol), previous diagnosis of major CVD (defined as disease of the circulatory system, arteries, and lymph, excluding hypertension), previous diagnosis of cancer, and familial history of CVD and cancer. Sleep (hours/day), physical activity (moderate to vigorous intensity (MVPA) minutes/day), and nutrition (Dietary Quality Score (DQS)) were included in the model as a joint term. The specific ranges for each exposure included sleep duration as 4.8-7.2 hours/day (low), 7.2-8.0 hours/day (medium), and 8.0-9.4 hours/day (high); MVPA measurements as 5-23 minutes/day (low), 23-42 minutes/day (medium), and 42-103 minutes/day (high); and diet quality using the DQS as 32.5-50.0 (low), 50.0-57.5 (medium), and 57.5-72.5 (high). Dashed blue lines separate tertiles MVPA and dashed black lines separate tertiles of sleep. Sleep (Slp); Low Diet Quality (LD); Medium Diet Quality (MD); High Diet Quality (HD).

**
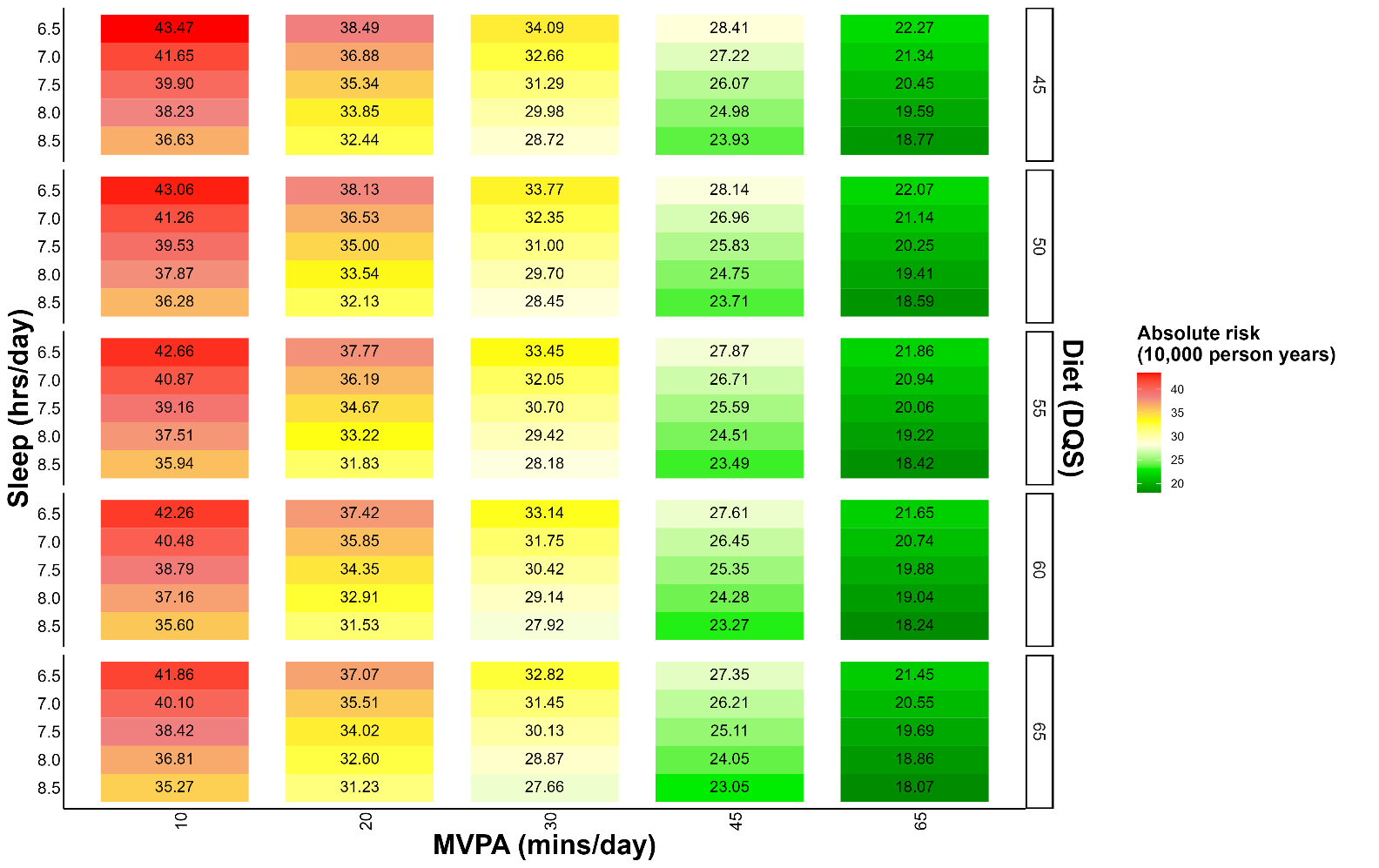
**

**Supplementary Figure 5: Absolute all-cause mortality risk associated with concurrent variations in sleep, MVPA, and dietary quality score (n = 59,078; events = 2,458)**

**Legend**: The correlogram displays changes in sleep (hours/day), physical activity (moderate to vigorous intensity (MVPA) minutes/day), and nutrition (Dietary Quality Score (DQS)) and absolute all-cause mortality risk as incidence per 10,000 person years with the reference being the 5^th^ percentile of sleep (5.5 hours/day), physical activity (7.3 minutes/day), and nutrition (36.9 DQS). Each square on the grid represents the absolute risk for all-cause mortality associated with a combination of behaviours, as defined by the x-axis (physical activity), y-axis (sleep), and z-axis (nutrition). The colour corresponds to the absolute risk where red indicates a higher risk of all-cause mortality and green indicates a lower risk of all-cause mortality. Model is adjusted for age, sex, ethnicity, smoking, education, Townsend deprivation index, alcohol, discretionary screen time (time spent watching TV or using the computer outside of work), light intensity physical activity, medication (blood pressure, insulin, and cholesterol), previous diagnosis of major CVD (defined as disease of the circulatory system, arteries, and lymph, excluding hypertension), previous diagnosis of cancer, and familial history of CVD and cancer.

**
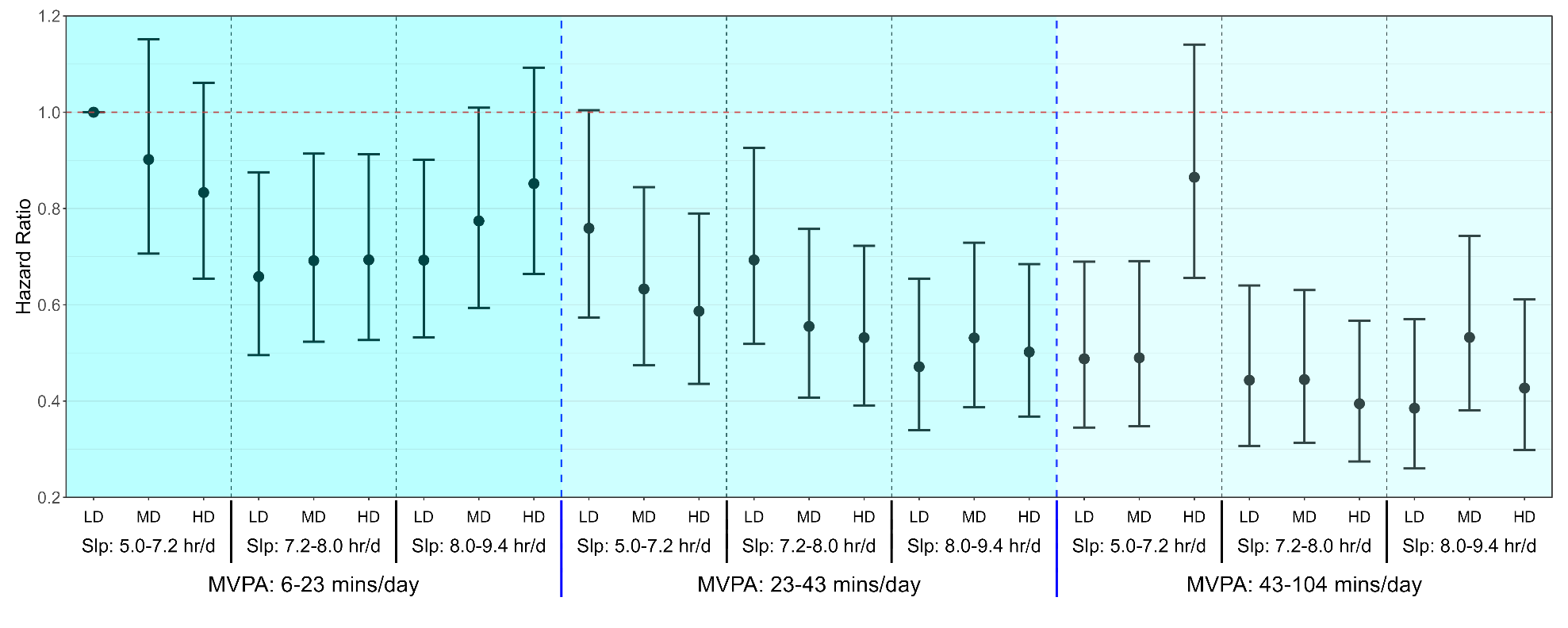
**

**Supplementary Figure 6: Multivariable-adjusted associations of combined Sleep, Physical Activity, and Nutrition with all-cause mortality excluding poor health individuals (n = 51,164; events = 1,887)**

**Legend**: Forest plot shows the SPAN associations with all-cause mortality after removing those with poor health status including, low BMI (<18.5), current smokers, self-reported poor health, and those with a frailty index score of >3. Model is adjusted for age, sex, ethnicity, smoking, education, Townsend deprivation index, alcohol, discretionary screen time (time spent watching TV or using the computer outside of work), light intensity physical activity, medication (blood pressure, insulin, and cholesterol), previous diagnosis of major CVD (defined as disease of the circulatory system, arteries, and lymph, excluding hypertension), previous diagnosis of cancer, and familial history of CVD and cancer. Sleep (hours/day), physical activity (moderate to vigorous intensity (MVPA) minutes/day), and nutrition (Dietary Quality Score, DQS) were included in the model as a joint term. The specific ranges for each exposure included sleep duration as 5.0-7.2 hours/day (low), 7.2-8.0 hours/day (medium), and 8.0-9.4 hours/day (high); MVPA measurements as 6-23 minutes/day (low), 23-42 minutes/day (medium), and 43-104 minutes/day (high); and diet quality using the DQS as 34.0-50.0 (low), 50.0-57.5 (medium), and 57.5-72.5 (high). The lowest tertiles for all three exposures (sleep, MVPA and DQS) was the referent group. Dashed blue lines separate tertiles MVPA and dashed black lines separate tertiles of sleep. Sleep (Slp); Low Diet Quality (LD); Medium Diet Quality (MD); High Diet Quality (HD).

**
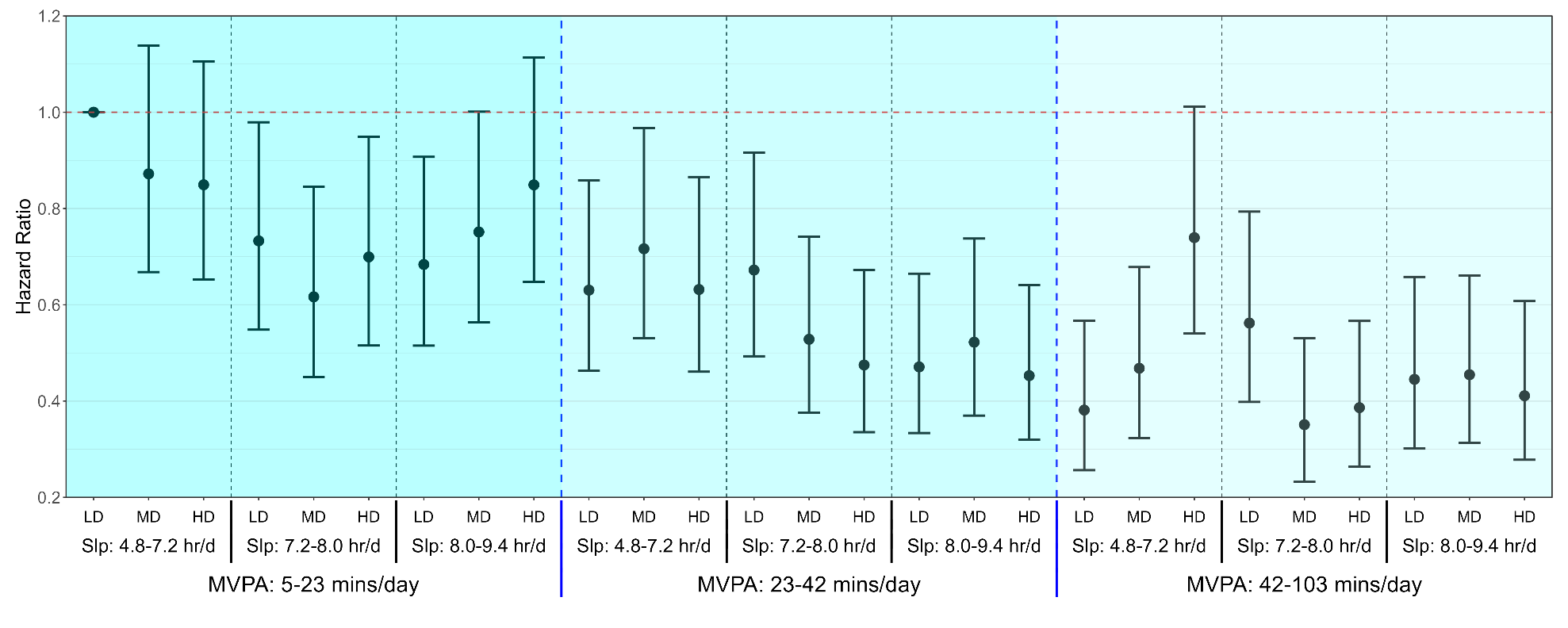
**

**Supplementary Figure 7: Multivariable-adjusted associations of combined Sleep, Physical Activity, and Nutrition with all-cause mortality excluding individuals with baseline CVD or cancer (n = 49,786; events = 1,637)**

**Legend**: Forest plot shows the SPAN associations with all-cause mortality after removing those with baseline cardiovascular disease (CVD) or cancer. Model is adjusted for age, sex, ethnicity, smoking, education, Townsend deprivation index, alcohol, discretionary screen time (time spent watching TV or using the computer outside of work), light intensity physical activity, medication (blood pressure, insulin, and cholesterol), and familial history of CVD and cancer. Sleep (hours/day), physical activity (moderate to vigorous intensity (MVPA) minutes/day), and nutrition (Dietary Quality Score (DQS)) were included in the model as a joint term. The specific ranges for each exposure included sleep duration as 5.0-7.2 hours/day (low), 7.2-8.0 hours/day (medium), and 8.0-9.4 hours/day (high); MVPA measurements as 6-23 minutes/day (low), 23-42 minutes/day (medium), and 43-104 minutes/day (high); and diet quality using the DQS as 34.0-50.0 (low), 50.0-57.5 (medium), and 57.5-72.5 (high). The lowest tertiles for all three exposures (sleep, MVPA and DQS) was the referent group. Dashed blue lines separate tertiles MVPA and dashed black lines separate tertiles of sleep. Sleep (Slp); Low Diet Quality (LD); Medium Diet Quality (MD); High Diet Quality (HD).

**
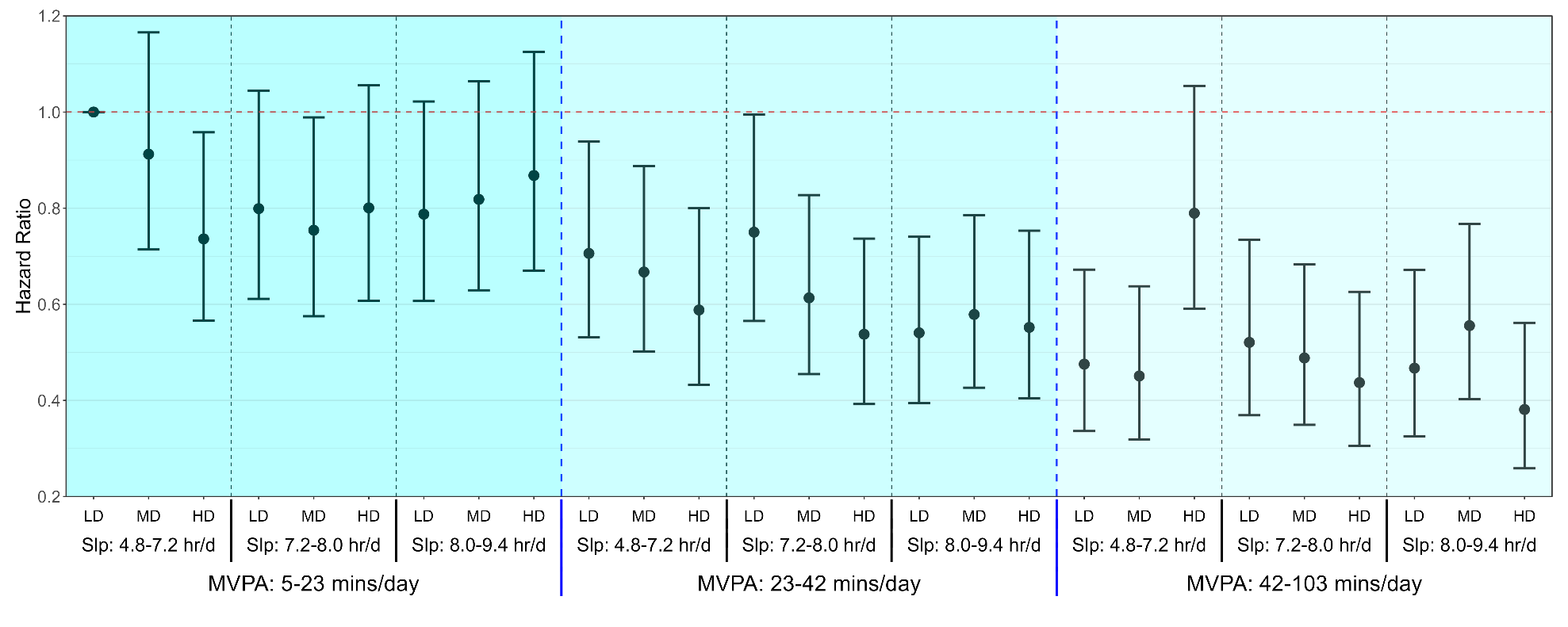
**

**Supplementary Figure 8: Multivariable-adjusted associations of combined Sleep, Physical Activity, and Nutrition with all-cause mortality excluding individuals with potentially sparse or outlier data (n = 48,670; events = 1,888)**

**Legend**: The associations above exclude individuals with potential sparse or outlier data (i.e., below 2.5 percentile or above 97.5 percentile) for sleep, physical activity, and nutrition. Model is adjusted for age, sex, ethnicity, smoking, education, Townsend deprivation index, alcohol, sedentary behaviour, light intensity physical activity, medication (blood pressure, insulin, and cholesterol), previous diagnosis of major CVD (defined as disease of the circulatory system, arteries, and lymph, excluding hypertension), previous diagnosis of cancer, familial history of CVD and cancer, and BMI. Sleep (hours/day), physical activity (moderate to vigorous intensity (MVPA) minutes/day), and nutrition (Dietary Quality Score (DQS)) were included in the model as a joint term. The specific ranges for each exposure included sleep duration as 4.8-7.2 hours/day (low), 7.2-8.0 hours/day (medium), and 8.0-9.4 hours/day (high); MVPA measurements as 5-23 minutes/day (low), 23-42 minutes/day (medium), and 42-103 minutes/day (high); and diet quality using the DQS as 32.5-50.0 (low), 50.0-57.5 (medium), and 57.5-72.5 (high). The lowest tertiles for all three exposures (sleep, MVPA and DQS) were considered the reference group. Dashed blue lines separate tertiles MVPA and dashed black lines separate tertiles of sleep. Sleep (Slp); Low Diet Quality (LD); Medium Diet Quality (MD); High Diet Quality (HD).

**
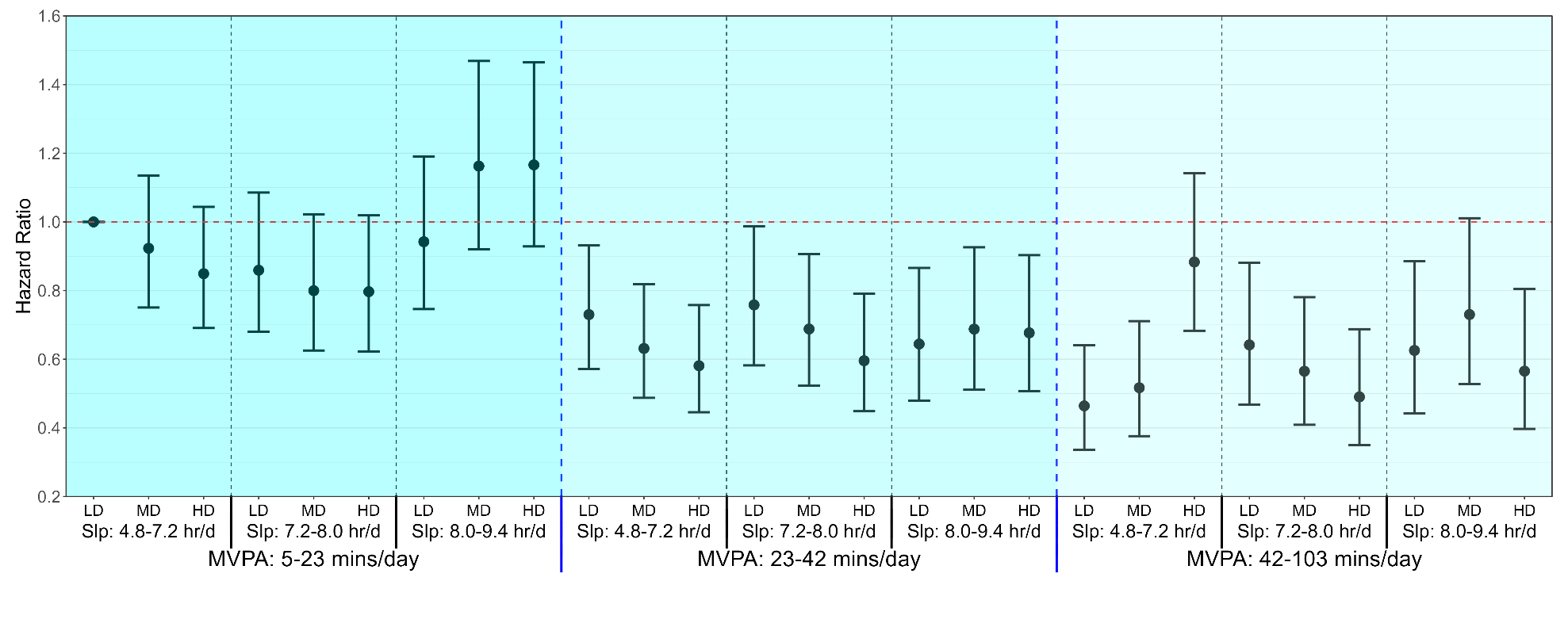
**

**Supplementary Figure 9: Multivariable-adjusted associations of combined Sleep, Physical Activity, and Nutrition with all-cause mortality adjusted for device measured sedentary behaviour (n = 59,078; events = 2,458)**

**Legend**: Model is adjusted for age, sex, ethnicity, smoking, education, Townsend deprivation index, alcohol, sedentary behaviour, light intensity physical activity, medication (blood pressure, insulin, and cholesterol), previous diagnosis of major CVD (defined as disease of the circulatory system, arteries, and lymph, excluding hypertension), previous diagnosis of cancer, familial history of CVD and cancer, and BMI. Sleep (hours/day), physical activity (moderate to vigorous intensity (MVPA) minutes/day), and nutrition (Dietary Quality Score (DQS)) were included in the model as a joint term. The specific ranges for each exposure included sleep duration as 4.8-7.2 hours/day (low), 7.2-8.0 hours/day (medium), and 8.0-9.4 hours/day (high); MVPA measurements as 5-23 minutes/day (low), 23-42 minutes/day (medium), and 42-103 minutes/day (high); and diet quality using the DQS as 32.5-50.0 (low), 50.0-57.5 (medium), and 57.5-72.5 (high). The lowest tertiles for all three exposures (sleep, MVPA and DQS) were considered the reference group. Dashed blue lines separate tertiles MVPA and dashed black lines separate tertiles of sleep. Sleep (Slp); Low Diet Quality (LD); Medium Diet Quality (MD); High Diet Quality (HD).


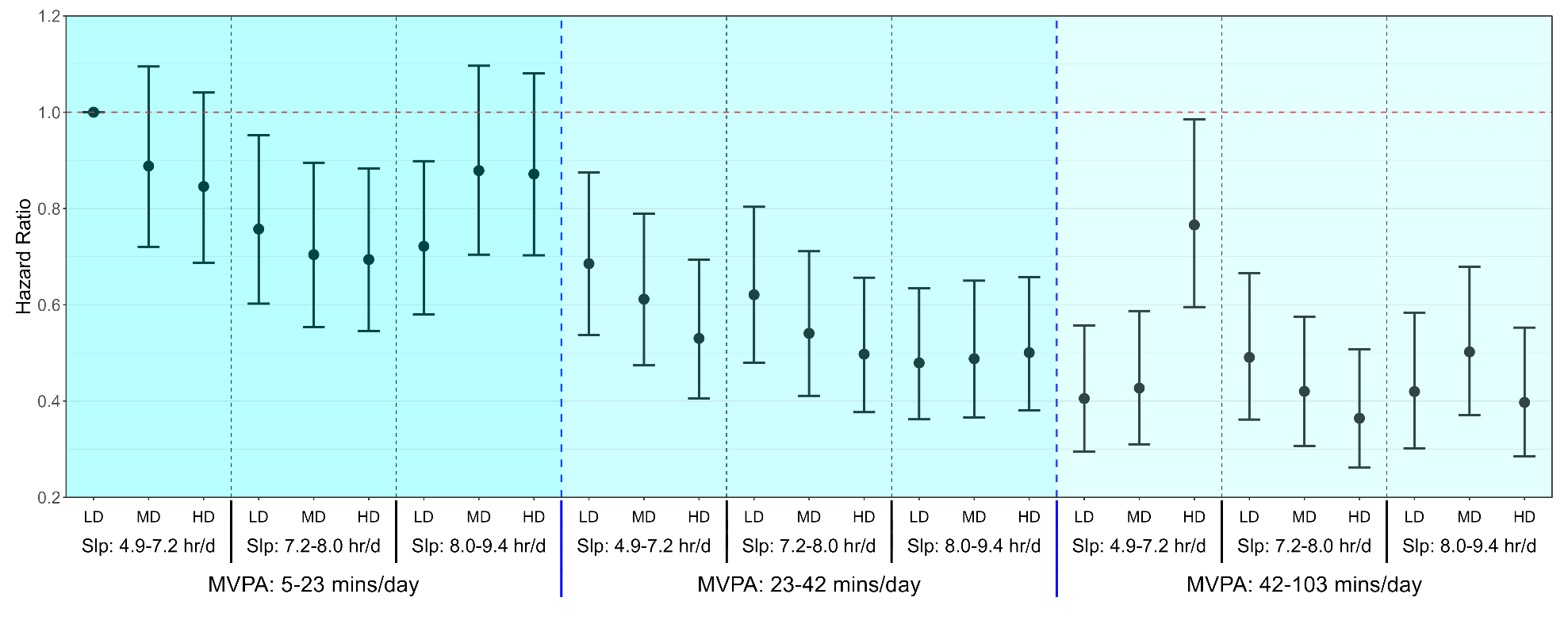


**Supplementary Figure 10: Multivariable-adjusted associations of combined Sleep, Physical Activity, and Nutrition with all-cause mortality adjusted for BMI (n = 58,363; events = 2,405)**

**Legend**: Model is adjusted for age, sex, ethnicity, smoking, education, Townsend deprivation index, alcohol, discretionary screen time (time spent watching TV or using the computer outside of work), light intensity physical activity, medication (blood pressure, insulin, and cholesterol), previous diagnosis of major CVD (defined as disease of the circulatory system, arteries, and lymph, excluding hypertension), previous diagnosis of cancer, familial history of CVD and cancer, and BMI. Sleep (hours/day), physical activity (moderate to vigorous intensity (MVPA) minutes/day), and nutrition (Dietary Quality Score (DQS)) were included in the model as a joint term. The specific ranges for each exposure included sleep duration as 4.8-7.2 hours/day (low), 7.2-8.0 hours/day (medium), and 8.0-9.4 hours/day (high); MVPA measurements as 5-23 minutes/day (low), 23-42 minutes/day (medium), and 42-103 minutes/day (high); and diet quality using the DQS as 32.5-50.0 (low), 50.0-57.5 (medium), and 57.5-72.5 (high). The lowest tertiles for all three exposures (sleep, MVPA and DQS) were considered the reference group. Dashed blue lines separate tertiles MVPA and dashed black lines separate tertiles of sleep. Sleep (Slp); Low Diet Quality (LD); Medium Diet Quality (MD); High Diet Quality (HD).


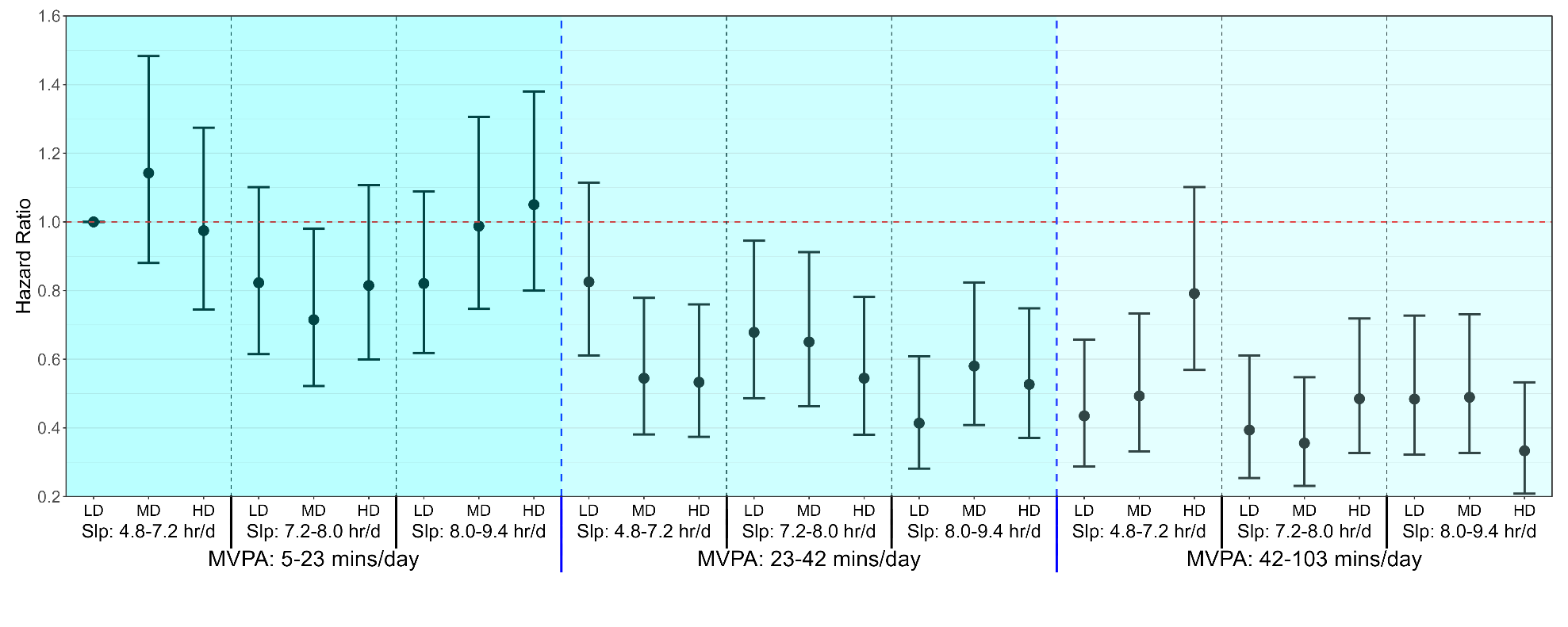


**Supplementary Figure 11: Multivariable-adjusted associations of combined Sleep, Physical Activity, and Nutrition with all-cause mortality adjusted for sleep characteristics (n = 37,475; events = 1,506)**

**Legend**: Model is adjusted for age, sex, ethnicity, smoking, education, Townsend deprivation index, alcohol, discretionary screen time (time spent watching TV or using the computer outside of work), light intensity physical activity, medication (blood pressure, insulin, and cholesterol), previous diagnosis of major CVD (defined as disease of the circulatory system, arteries, and lymph, excluding hypertension), previous diagnosis of cancer, familial history of CVD and cancer, insomnia, snoring, chronotype (morning/evening person), and daytime sleepiness. Sleep (hours/day), physical activity (moderate to vigorous intensity (MVPA) minutes/day), and nutrition (Dietary Quality Score (DQS)) were included in the model as a joint term. The specific ranges for each exposure included sleep duration as 4.8-7.2 hours/day (low), 7.2-8.0 hours/day (medium), and 8.0-9.4 hours/day (high); MVPA measurements as 5-23 minutes/day (low), 23-42 minutes/day (medium), and 42-103 minutes/day (high); and diet quality using the DQS as 32.5-50.0 (low), 50.0-57.5 (medium), and 57.5-72.5 (high). The lowest tertiles for all three exposures (sleep, MVPA and DQS) were considered the reference group. Dashed blue lines separate tertiles MVPA and dashed black lines separate tertiles of sleep. Sleep (Slp); Low Diet Quality (LD); Medium Diet Quality (MD); High Diet Quality (HD).


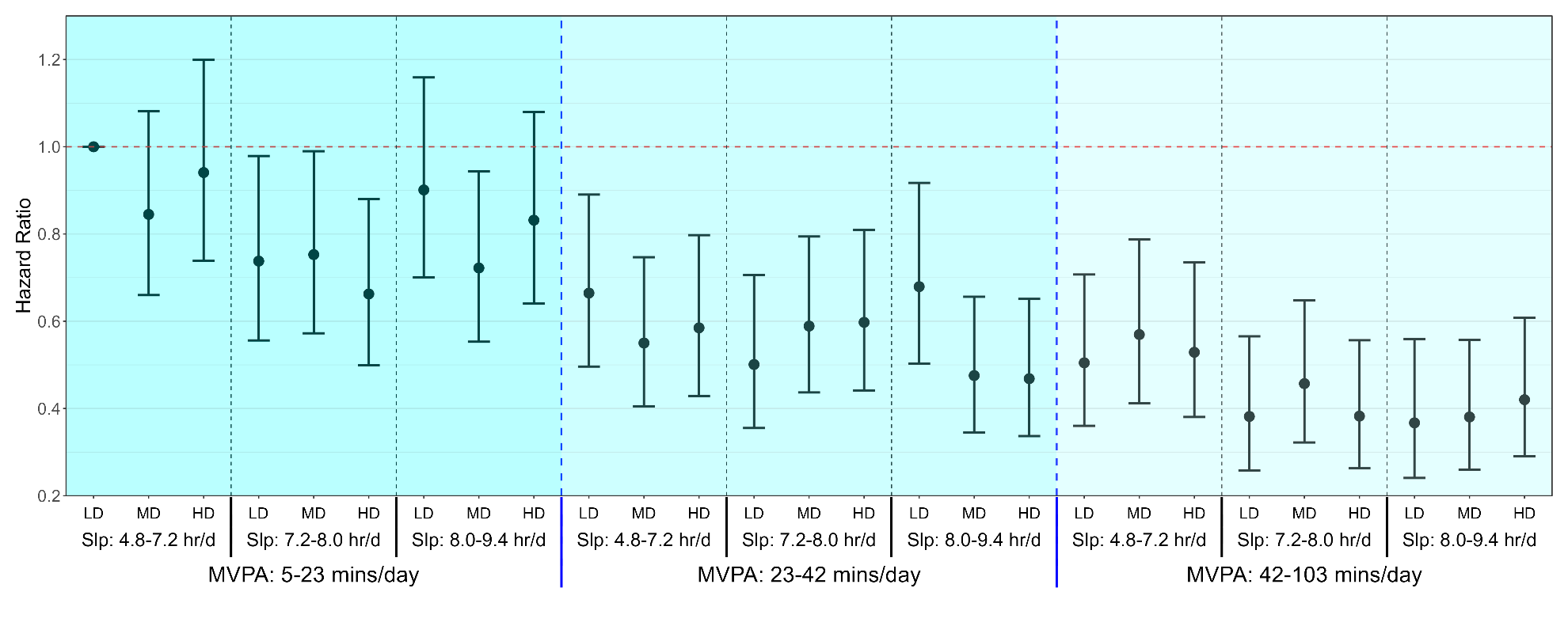


**Supplementary Figure 12: Multivariable-adjusted associations of combined Sleep, Physical Activity, and Nutrition with all-cause mortality using the proportion of ultra-processed food (n = 41,936; events = 1,758)**

**Legend**: Model is adjusted for age, sex, ethnicity, smoking, education, Townsend deprivation index, alcohol, discretionary screen time (time spent watching TV or using the computer outside of work), light intensity physical activity, medication (blood pressure, insulin, and cholesterol), previous diagnosis of major CVD (defined as disease of the circulatory system, arteries, and lymph, excluding hypertension), previous diagnosis of cancer, familial history of CVD and cancer. From 2009-2012, dietary data was also collected using 1-4 separate 24-hour dietary recall for a subgroup of participants (n = 211,031)1. Diet quality was defined as the percentage of dietary ultra-processed food where higher diet quality had a lower proportion of ultra-processed food in the diet. Sleep (hours/day), physical activity (moderate to vigorous intensity (MVPA) minutes/day), and nutrition (ultra-processed food intake, % of total diet by weight) were included in the model as a joint term. The specific ranges for each exposure included sleep duration as 4.8-7.2 hours/day (low), 7.2-8.0 hours/day (medium), and 8.0-9.4 hours/day (high); MVPA measurements as 5-23 minutes/day (low), 23-42 minutes/day (medium), and 42-103 minutes/day (high); and diet quality using the proportion of ultra-processed food as 21.5-100.0 (low), 13.2-21.5 (medium), and 0.0-13.2% (high). The lowest tertiles for all three exposures (sleep, MVPA and DQS) were considered the reference group. Dashed blue lines separate tertiles MVPA and dashed black lines separate tertiles of sleep. Sleep (Slp); Low Diet Quality (LD); Medium Diet Quality (MD); High Diet Quality (HD).


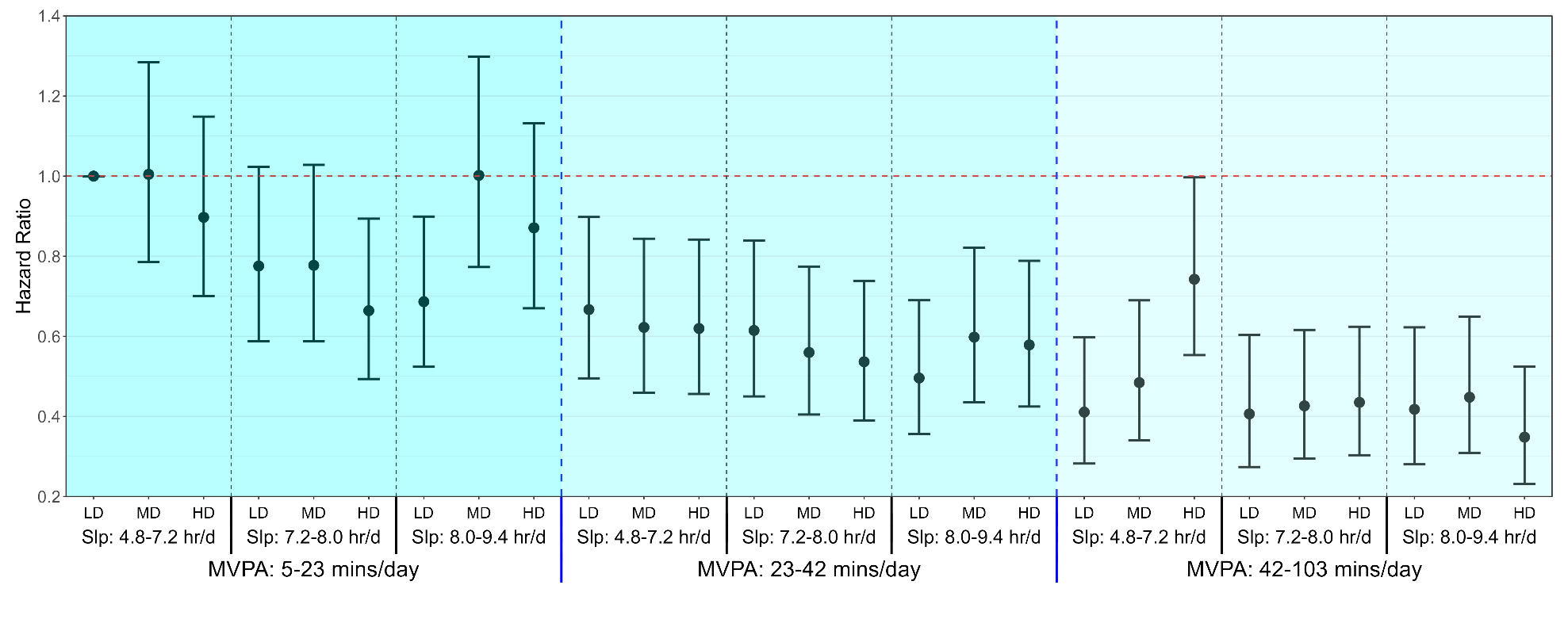


**Supplementary Figure 13: Multivariable-adjusted associations of combined Sleep, Physical Activity, and Nutrition with all-cause mortality adjusted for total energy intake (n = 42,990; 1,758 events)**

**Legend**: Model is adjusted for age, sex, ethnicity, smoking, education, Townsend deprivation index, alcohol, discretionary screen time (time spent watching TV or using the computer outside of work), light intensity physical activity, medication (blood pressure, insulin, and cholesterol), previous diagnosis of major CVD (defined as disease of the circulatory system, arteries, and lymph, excluding hypertension), previous diagnosis of cancer, familial history of CVD and cancer, and total energy intake. From 2009-2012, dietary data was also collected using 1-4 separate 24-hour dietary recall for a subgroup of participants (n = 211,031)1. Energy intake outliers (<800 or >4200 kcal/ per day) for men and (<600 or >3500 kcal per day) for women were also excluded from this sample[54]. Sleep (hours/day), physical activity (moderate to vigorous intensity (MVPA) minutes/day), and nutrition (Dietary Quality Score (DQS)) were included in the model as a joint term. The specific ranges for each exposure included sleep duration as 4.8-7.2 hours/day (low), 7.2-8.0 hours/day (medium), and 8.0-9.4 hours/day (high); MVPA measurements as 5-23 minutes/day (low), 23-42 minutes/day (medium), and 42-103 minutes/day (high); and diet quality using the DQS as 32.5-50.0 (low), 50.0-57.5 (medium), and 57.5-72.5 (high). The lowest tertiles for all three exposures (sleep, MVPA and DQS) were considered the reference group. Dashed blue lines separate tertiles MVPA and dashed black lines separate tertiles of sleep. Sleep (Slp); Low Diet Quality (LD); Medium Diet Quality (MD); High Diet Quality (HD).

**Supplementary Table 1: Diet quality score index for food-frequency questionnaire dietary data**

| **Food components** | **UK Biobank field ID** | **Amount per serving** | **Criteria for maximum score (10)** | **Criteria for minimum score (0)** |
| --- | --- | --- | --- | --- |
| Fruit | 1309 (pieces fresh fruit/day) 1319 (pieces dried fruit/day) | 1309 – 1 piece  1319 – 5 pieces | ≥3 servings/day | 0 servings/day |
| Vegetable | 1289 (tablespoons cooked vegetables/day)  1299 (salad/raw vegetables/day) | 3 heaped tablespoons | ≥3 servings/day | 0 servings/day |
| Whole grains | 1438, 1448 (wholemeal/wholegrain bread slices/week)  1458, 1468 (bran/oat/muesli cereal) | 1438/1448 – 1 slice/day 1458/1468 – 1 bowl/day | ≥3 servings/day | 0 servings/day |
| Fish | 1329 (oily fish/week)  1339 (non-oily fish/week) | Once/week | ≥2 servings/week | 0 servings/week |
| Dairy | 1408 (cheese/week)  1418 (milk type) | 1408 – 1 piece/day  1418 – 1 glass/day if consumption of any type of milk | ≥2 servings/day | 0 servings/day |
| Vegetable oils | 1428 (Flora Pro-Active/Benecol spread)  2654 (Flora Pro-Active/Benecol, soft margarine -, olive oil based -, polyunsaturated/sunflower oil based -, other low/reduced fat spread)  1438 (bread slices/week) | 1 serving/day if in combination with eating at least 2 slices of bread (ID 1438) | ≥2 servings/day | 0 servings/day |
| Refined grains | 1438, 1448 (white, brown, other bread slices/week) 1458, 1468 (biscuit, other cereals/week) | 1438/1448 – 1 slice/day 1458/1468 – 1 bowl/day | 0 servings/day | >2 servings/day |
| Processed meats | 1349 (processed meat/week or daily)  3680 (age when last ate meat) | 1349 – 1 piece/day  3680 – 0 pieces/day if indicated having never eaten meat | 0 serving/week | >1 serving/week |
| Unprocessed red meats | 1369 (beef/week or day)  1379 (lamb or mutton/week or day)  1389 (pork/week or day)  3680 (age when last ate meat) | 1359-1389 – once/week 3680 – 0 pieces/day if indicated having never eaten meat | 0 serving/week | >2 serving/week |
| Sugar-sweetened beverages | 6144 (never consumes drinks containing sugar) | 0 servings | Don’t drink | Drink |

Diet quality score information is adapted from previously established work by Zhuang *et al*. Diabetes Care[40]. Intermediate intake for each dietary component were scored relative to minimum to maximum intake of each food component. The formula for intermediate intakes of adequacy components is described as: component score = (maximum score / (Amax - Amin))*(X - Amin) and for moderate components (refined grains, processed meat, and unprocessed red meat) component score = (maximum score - maximum score / (Amax - Amin))*(X - Amin). The food frequency questionnaire demonstrated moderate reproducibility for food groups (Intraclass Correlation Coefficient (ICC): 0.48-0.66) and modest agreement with alternative dietary intake measures from the 24-hour recall (ICC: 0.38-0.63). This level of agreement and reproducibility is comparable to previous prospective observational studies[41-43]. The food frequency questionnaire has also been validated against the 24-hour dietary recall using objective biomarkers as the standard[38].

|  |  | **Nutrition Low** | **Nutrition Medium** | **Nutrition High** |
| --- | --- | --- | --- | --- |
| **MVPA Low** | **Sleep Low** | n = 2542 (Events = 203) | n = 2234 (Events = 163) | n = 2205 (Events = 166) |
|  | **Sleep Medium** | n = 2178 (Events = 116) | n = 1982 (Events = 100) | n = 1861 (Events = 100) |
|  | **Sleep High** | n = 2458 (Events = 138) | n = 2136 (Events = 138) | n = 2097 (Events = 152) |
| **MVPA Medium** | **Sleep Low** | n = 2312 (Events = 98) | n = 2116 (Events = 83) | n = 1945 (Events = 77) |
|  | **Sleep Medium** | n = 2298 (Events = 85) | n = 2241 (Events = 75) | n = 2092 (Events = 69) |
|  | **Sleep High** | n = 2339 (Events = 67) | n = 2180 (Events = 66) | n = 2169 (Events = 73) |
| **MVPA High** | **Sleep Low** | n = 2271 (Events = 48) | n = 2062 (Events = 50) | n = 2006 (Events = 92) |
|  | **Sleep Medium** | n = 2407 (Events = 55) | n = 2386 (Events = 52) | n = 2247 (Events = 46) |
|  | **Sleep High** | n = 2113 (Events = 46) | n = 2127 (Events = 56) | n = 2074 (Events = 44) |

**Supplementary Table 2: Sample size and all-cause mortality events for each Sleep, Physical Activity, and Nutrition category**

The sample size and number of all-cause mortality events for each Sleep, Physical Activity, and Nutrition group is detailed above (n = 59,078; events = 2,458). Participants were grouped by Sleep, Physical Activity, and Nutrition exposure tertiles (i.e., low, moderate, and high) which equated to a joint exposure of 27 separate groups for all three behaviours. The specific ranges for each exposure included sleep duration as 4.8-7.2 hours/day (low), 7.2-8.0 hours/day (medium), and 8.0-9.4 hours/day (high); moderate to vigorous physical activity (MVPA) measurements as 5-23 minutes/day (low), 23-42 minutes/day (medium), and 42-103 minutes/day (high); and diet quality using the DQS as 32.5-50.0 (low), 50.0-57.5 (medium), and 57.5-72.5 (high).

**Supplementary Table 3: Covariate Definitions**

| **Variable** | **Definition** | **UK Biobank field ID (if applicable)** |
| --- | --- | --- |
| Age | Categorical (4) | 34, 52, accelerometer date-timestamp |
| Sex | Female/Male | 31 |
| Ethnicity | White/Others | 21000 |
| Education | College/University; A/AS level; O levels; CSE; NVQ/HND/HNC; other | 6138 |
| Smoking status | Never, past, current | 20116 |
| Alcohol consumption | Units/day | 20403 |
| Light intensity physical activity | Standing utilitarian movements, slow walking (<3 METs) | Derived from accelerometer data |
| Discretionary screen-time | Time spent/day watching TV and using a computer outside of work | 1070, 1080 |
| Townsend deprivation | Categorical (5) | 22189 |
| Use of cholesterol medication | Yes/No | 6177, 6153 |
| Use of blood pressure medication | Yes/No | 6177, 6153 |
| Use of diabetes medication | Yes/No | 6177, 6153 |
| Previous CVD | Identified by self-report and cancer registry. Defined as disease of the circulatory system, arteries, and lymph, excluding hypertension | 20002, 41270 |
| Previous cancer | Identified by self-report and hospitalisation | 20001, 100092 |
| Familial history of CVD | Self-reporter mother of father diagnosed with heart disease or stroke | 20107, 20110 |
| Familial history of cancer | Self-reporter mother of father diagnosed with cancer | 20107, 20110 |
| High frailty scale | Categorical (yes/no); high frailty indicates a score of ≥3 on a 0 to 5 | 2306, 120107, 2624, 1011, 3637, 991, 971, 924, 46, 47 |
| Body mass index | Continuous; kilogram/meter^2^ | 23104 |
| Total energy intake | Continuous, kcal/day | 26002 |
| Morning/evening person (chronotype) | Categorical (definitely a ‘morning’ person; more a ‘morning’ person than ‘evening’ person; more an ‘evening’ person than ‘morning’ person; definitely an ‘evening’ person) | 1180 |
| Insomnia | Categorical (never/rarely; sometimes; usually) | 1200 |
| Snoring | Categorical (yes/no) | 1210 |
| Daytime sleepiness | Categorical (never/rarely; sometimes; often) | 1220 |

Additional detail is available online at https://biobank.ndph.ox.ac.uk/showcase/.

**Supplementary Table 4: Model variance inflation factors for combined SPAN behaviours**

| **Primary Model with Combined SPAN Behaviours** | |
| --- | --- |
| **Variable** | **Variance inflation factor (VIF)** |
| Sleep, moderate to vigorous physical activity, and nutrition (combined variable) | 1.28 |
| Age (self-report) | 1.17 |
| Sex (self-report) | 1.17 |
| Ethnicity (self-report) | 1.03 |
| Smoking (self-report) | 1.10 |
| Alcohol (self-report) | 1.15 |
| Education (self-report) | 1.08 |
| Socioeconomic status (self-report) | 1.06 |
| Light physical activity (accelerometry derived) | 1.14 |
| Previous CVD (self-report) | 1.15 |
| Previous cancer (self-report) | 1.03 |
| Familial history of CVD (self-report) | 1.01 |
| Familial history of cancer (self-report) | 1.01 |
| Discretionary screen time (self-report) | 1.09 |
| Medication (self-report) | 1.21 |
| **Model Adjusted for Accelerometry Derived Sedentary Behaviour** | |
| **Variable** | **Variance inflation factor (VIF)** |
| Sleep, moderate to vigorous physical activity, and nutrition (combined variable) | 2.43 |
| Age (self-report) | 1.19 |
| Sex (self-report) | 1.18 |
| Ethnicity (self-report) | 1.03 |
| Smoking (self-report) | 1.10 |
| Alcohol (self-report) | 1.15 |
| Education (self-report) | 1.07 |
| Socioeconomic status (self-report) | 1.06 |
| Light physical activity (accelerometry derived) | 1.68 |
| Previous CVD (self-report) | 1.15 |
| Previous cancer (self-report) | 1.03 |
| Familial history of CVD (self-report) | 1.03 |
| Familial history of cancer (self-report) | 1.01 |
| Sedentary behaviour (accelerometry derived) | 2.81 |
| Medication (self-report) | 1.21 |

The table provides the variance inflation factor (VIF) for each covariate in the primary analytical model (combined SPAN behaviours) adjusted for self-reported discretionary screen time and the sensitivity model adjusted for accelerometry derived sedentary behaviour. VIF values measure multicollinearity among the exposure variables, with a value of 1 indicating no correlation with other predictors. Higher values suggest increasing multicollinearity, with values greater than 5 indicating potentially problematic multicollinearity[49].

**Supplementary Table 5: Model variance inflation factors for individual SPAN behaviours**

| **Primary Model with Individual SPAN Behaviours** | |
| --- | --- |
| **Variable** | **Variance inflation factor (VIF)** |
| Sleep (accelerometry derived) | 1.03 |
| Moderate to vigorous physical activity (accelerometry derived) | 1.21 |
| Nutrition (self-report) | 1.04 |
| Age (self-report) | 1.18 |
| Sex (self-report) | 1.17 |
| Ethnicity (self-report) | 1.03 |
| Smoking (self-report) | 1.10 |
| Alcohol (self-report) | 1.15 |
| Education (self-report) | 1.08 |
| Socioeconomic status (self-report) | 1.05 |
| Light physical activity (accelerometry derived) | 1.15 |
| Previous CVD (self-report) | 1.15 |
| Previous cancer (self-report) | 1.03 |
| Familial history of CVD (self-report) | 1.03 |
| Familial history of cancer (self-report) | 1.01 |
| Discretionary screen time (self-report) | 1.09 |
| Medication (self-report) | 1.22 |
| **Model Adjusted for Accelerometry Derived Sedentary Behaviour** | |
| **Variable** | **Variance inflation factor (VIF)** |
| Sleep (accelerometry derived) | 2.25 |
| Moderate to vigorous physical activity (accelerometry derived) | 1.56 |
| Nutrition (self-report) | 1.04 |
| Age (self-report) | 1.19 |
| Sex (self-report) | 1.19 |
| Ethnicity (self-report) | 1.03 |
| Smoking (self-report) | 1.10 |
| Alcohol (self-report) | 1.15 |
| Education (self-report) | 1.07 |
| Socioeconomic status (self-report) | 1.06 |
| Light physical activity (accelerometry derived) | 1.81 |
| Previous CVD (self-report) | 1.15 |
| Previous cancer (self-report) | 1.03 |
| Familial history of CVD (self-report) | 1.03 |
| Familial history of cancer (self-report) | 1.01 |
| Sedentary behaviour (accelerometry derived) | 3.71 |
| Medication (self-report) | 1.21 |

The table provides the variance inflation factor (VIF) for each covariate in the primary analytical model (individual SPAN behaviours) adjusted for self-reported discretionary screen time and the sensitivity model adjusted for accelerometry derived sedentary behaviour. VIF values measure multicollinearity among the exposure variables, with a value of 1 indicating no correlation with other predictors. Higher values suggest increasing multicollinearity, with values greater than 5 indicating potentially problematic multicollinearity[49].

**Supplementary Table 6: NOVA classification of food groups for 24-hour dietary recall data**

| **NOVA classification level** | **UK Biobank field ID (if applicable)** |
| --- | --- |
| Level four | Added sugars and preserves (26064), Animal fat spread lower fat (26062), Animal fat spread normal (26063), Biscuit cereal (26075), Biscuits (26068), Bran cereal (26076), Breaded/battered chicken (26069), Breaded/battered fish (26070), Chocolate confectionery (26080), Cream (26154), Fried/roast potatoes (26119), Low/non sugar sugar-sweetened beverages (26126), Mashed potatoes (26120), Meat substitutes - soy (26137), Meat substitutes - vegetarian (26145), Milk-based and powdered drinks (26087), Milk-dairy desserts (26084), Mixed bread brown and seeded (26071), Muesli (26105), Nut-based spreads (26106), Other cereal (sugar) (26079), Other desserts and cakes and pastries (26085), Other sweets (26140), Pizza (26116), Plant-based spread lower fat (26111), Plant-based spread normal (26112), Processed meat (26122), Samosa, pakora (26128), Sauces and condiments (high fat) (26129), Sauces and condiments (low fat) (26130), Savoury crackers (26083), Savoury snacks (26134), Soy desserts and yogurt (26086), Sugar-sweetened beverages and other sugary drinks (26127), Sushi (26139), Vegetable dips (26144) |
| Level three | High fat cheese (26099), Medium and low fat cheese (26103), White fish and tinned tuna (26149), White bread (26073), Wholemeal bread (26074), Other bread (26072) |
| Level two | Grain dishes - added fat (26097), Olive oil (drizzling/dunking) (26110) |
| Level one | Allium vegetables (26065), Apples and pears (26089), Beef (26066), Berries (26090), Citrus (26091), Coffee, caffeinated (26081), Coffee, decaffeinated (26082), Dried fruit (26092), Egg and egg dishes (26088), Fruit juice (26095), Green leafy/cabbages (26098), Lamb (26100), Legumes and pulses (26101), Oat cereal (non sugar) (26077), Oat cereal (sugar) (26078), Low fat yogurt (26102), Full fat yogurt (26096), Oily fish (26109), Other fruit (26093), Other meat, offal (26104), Other vegetables, including mushrooms, fruiting and mixed vegetables (26146), Peas and sweetcorn (26115), Pork (26117), Potatoes and sweet potatoes (baked/boiled) (26118), Poultry (26121), Raw salad (26123), Root vegetables (26125), Salted nuts and seeds (26108), Semi skimmed milk (26131), Rice/oat milk (26124), Shellfish (26132), Skimmed milk and cholesterol-lowering milk (26133), Soups (26135), Soy milk (26136), Stewed fruit (26094), Tea (26141), Tea, decaffeinated (26142), Tomatoes (26143), Unsalted nuts and seeds (26107), White pasta and rice (26113), Whole milk (26150), Wholemeal pasta, brown rice and other wholegrains (26114) |

From 2009-2012, dietary data was also collected using 1-4 separate 24-hour dietary recall for a subgroup of participants (n = 211,031)[54]. Additional detail on reproducibility and agreement between FFQ and the 24-hour dietary recall has been published elsewhere[37, 53]. Food groups in each NOVA classification level were reported as the average weight (gram/day) from the 24-hour dietary recalls. Ultra-processed food intake was defined as the percentage of level four NOVA food groups relative to the average reported total food weight. All food categories and the definition of ultra-processed food intake were determined using a previously established method[51, 52].

**Supplementary Table 7: STROBE Statement**

|  | Item No | Recommendation | Page No |
| --- | --- | --- | --- |
| **Title and abstract** | 1 | (*a*) Indicate the study’s design with a commonly used term in the title or the abstract | 1 |
|  |  | (*b*) Provide in the abstract an informative and balanced summary of what was done and what was found | 2 |
| Introduction | | | |
| Background/rationale | 2 | Explain the scientific background and rationale for the investigation being reported | 3 |
| Objectives | 3 | State specific objectives, including any prespecified hypotheses | 4-5 |
| Methods | | | |
| Study design | 4 | Present key elements of study design early in the paper |  |
| Setting | 5 | Describe the setting, locations, and relevant dates, including periods of recruitment, exposure, follow-up, and data collection | 5 |
| Participants | 6 | (*a*) Give the eligibility criteria, and the sources and methods of selection of participants. Describe methods of follow-up | 5 |
|  |  | (*b*) For matched studies, give matching criteria and number of exposed and unexposed | - |
| Variables | 7 | Clearly define all outcomes, exposures, predictors, potential confounders, and effect modifiers. Give diagnostic criteria, if applicable | 6 |
| Data sources/ measurement | 8* | For each variable of interest, give sources of data and details of methods of assessment (measurement). Describe comparability of assessment methods if there is more than one group | 5 |
| Bias | 9 | Describe any efforts to address potential sources of bias | 7-8 |
| Study size | 10 | Explain how the study size was arrived at | 5, 8 |
| Quantitative variables | 11 | Explain how quantitative variables were handled in the analyses. If applicable, describe which groupings were chosen and why | 6 |
| Statistical methods | 12 | (*a*) Describe all statistical methods, including those used to control for confounding | 6-8 |
|  |  | (*b*) Describe any methods used to examine subgroups and interactions | 8 |
|  |  | (*c*) Explain how missing data were addressed | 5 |
|  |  | (*d*) If applicable, explain how loss to follow-up was addressed | 5 |
|  |  | (*e*) Describe any sensitivity analyses | 8 |
| Results | | |  |
| Participants | 13* | (a) Report numbers of individuals at each stage of study—eg numbers potentially eligible, examined for eligibility, confirmed eligible, included in the study, completing follow-up, and analysed | 5 |
|  |  | (b) Give reasons for non-participation at each stage | Supplemental figure 1 |
|  |  | (c) Consider use of a flow diagram | Supplemental figure 1 |
| Descriptive data | 14* | (a) Give characteristics of study participants (eg demographic, clinical, social) and information on exposures and potential confounders | Table 1 |
|  |  | (b) Indicate number of participants with missing data for each variable of interest | Supplemental figure 1 |
|  |  | (c) Summarise follow-up time (eg, average and total amount) | Table 1 |
| Outcome data | 15* | Report numbers of outcome events or summary measures over time | 8 |

| Main results | 16 | (*a*) Give unadjusted estimates and, if applicable, confounder-adjusted estimates and their precision (eg, 95% confidence interval). Make clear which confounders were adjusted for and why they were included | 8-9 |
| --- | --- | --- | --- |
|  |  | (*b*) Report category boundaries when continuous variables were categorized | 8-9 |
|  |  | (*c*) If relevant, consider translating estimates of relative risk into absolute risk for a meaningful time period | 10 |
| Other analyses | 17 | Report other analyses done—eg analyses of subgroups and interactions, and sensitivity analyses | 11 |
| Discussion | | | |
| Key results | 18 | Summarise key results with reference to study objectives | 12 |
| Limitations | 19 | Discuss limitations of the study, taking into account sources of potential bias or imprecision. Discuss both direction and magnitude of any potential bias | 14-15 |
| Interpretation | 20 | Give a cautious overall interpretation of results considering objectives, limitations, multiplicity of analyses, results from similar studies, and other relevant evidence | 15 |
| Generalisability | 21 | Discuss the generalisability (external validity) of the study results | 15 |
| Other information | | | |
| Funding | 22 | Give the source of funding and the role of the funders for the present study and, if applicable, for the original study on which the present article is based | 16 |

*Give information separately for exposed and unexposed groups.

**Note:** An Explanation and Elaboration article discusses each checklist item and gives methodological background and published examples of transparent reporting. The STROBE checklist is best used in conjunction with this article (freely available on the Web sites of PLoS Medicine at http://www.plosmedicine.org/, Annals of Internal Medicine at http://www.annals.org/, and Epidemiology at http://www.epidem.com/). Information on the STROBE Initiative is available at http://www.strobe-statement.org.

| **Model term** | **Coefficient** | **Standard error** | | **P-value** |
| --- | --- | --- | --- | --- |
| Sleep | -0.52 | 0.04 | | 0.004 |
| Physical Activity | -0.11 | 0.16 | | 0.001 |
| Nutrition | -0.07 | 0.02 | | 0.002 |
| Physical Activity*Sleep | 0.01 | 0.005 | | 0.01 |
| Physical Activity*Nutrition | 0.002 | 0.0007 | | 0.004 |
| Sleep*Nutrition | 0.009 | 0.003 | | 0.004 |
| Physical Activity*Nutrition*Sleep | -0.0003 | 0.0001 | | 0.005 |
|  | | | | |
| **Index** | **Estimate** | | **95% CI** | |
| RERI | 0.06 | | 0.004, 0.13 | |
| AP | 0.12 | | 0.01-0.39 | |
| S | 0.89 | | 0.84-0.97 | |

**Supplementary Table 8: Relative excess risk due to interaction**

The table above shows the individual and interactive model terms of Sleep, Physical Activity, and Nutrition for all-cause mortality with the reference being the 5th percentile of sleep (5.5 hours/day), physical activity (7.3 minutes/day), and nutrition (36.9 DQS). To test for interactive and synergistic effects, we calculated the relative excess risk due to interaction (RERI), attributable proportion due to interaction (AP), and the synergistic effects index (S)[50]. These tests provide insight into the contribution of synergistic interactions between exposures where an RERI or AP of 0 and an S value of 1 denote no interaction effect.
